# Reduced on-line speech gesture integration during multimodal language processing in adults with moderate-severe traumatic brain injury: Evidence from eye-tracking

**DOI:** 10.1016/j.cortex.2024.08.008

**Published:** 2024-10-22

**Authors:** Sharice Clough, Sarah Brown-Schmidt, Sun-Joo Cho, Melissa C. Duff

**Affiliations:** a Department of Hearing and Speech Sciences, Vanderbilt University Medical Center, Nashville, Tennessee, USA; b Multimodal Language Department, Max Planck Institute for Psycholinguistics, Nijmegen, The Netherlands; c Department of Psychology and Human Development, Vanderbilt University, Nashville, Tennessee, USA

**Keywords:** Traumatic brain injury, Speech-gesture integration, Multimodal communication, Eye-tracking, Language processing

## Abstract

**Background:**

Language is multimodal and situated in rich visual contexts. Language is also incremental, unfolding moment-to-moment in real time, yet few studies have examined how spoken language interacts with gesture and visual context during multimodal language processing. Gesture is a rich communication cue that is integrally related to speech and often depicts concrete referents from the visual world. Using eye-tracking in an adapted visual world paradigm, we examined how participants with and without moderate-severe traumatic brain injury (TBI) use gesture to resolve temporary referential ambiguity.

**Methods:**

Participants viewed a screen with four objects and one video. The speaker in the video produced sentences (e.g., “The girl will eat the very good sandwich”), paired with either a meaningful gesture (e.g., sandwich-holding gesture) or a meaningless grooming movement (e.g., arm scratch) at the verb “will eat.” We measured participants’ gaze to the target object (e.g., sandwich), a semantic competitor (e.g., apple), and two unrelated distractors (e.g., piano, guitar) during the critical window between movement onset in the gesture modality and onset of the spoken referent in speech.

**Results:**

Both participants with and without TBI were more likely to fixate the target when the speaker produced a gesture compared to a grooming movement; however, relative to non-injured participants, the effect was significantly attenuated in the TBI group.

**Discussion:**

We demonstrated evidence of reduced speech-gesture integration in participants with TBI relative to non-injured peers. This study advances our understanding of the communicative abilities of adults with TBI and could lead to a more mechanistic account of the communication difficulties adults with TBI experience in rich communication contexts that require the processing and integration of multiple co-occurring cues. This work has the potential to increase the ecological validity of language assessment and provide insights into the cognitive and neural mechanisms that support multimodal language processing.

## 1. Introduction

Language is multimodal, containing both speech and gesture. Gesture is a form of visual language that enriches everyday communication. Although gestures occur simultaneously with speech, they often communicate unique information, particularly about visuospatial descriptions and actions (Alibali, 2005; Feyereisen & Havard, 1999; Hostetter & Alibali, 2019; Melinger & Levelt, 2004). Gestures that meaningfully depict aspects of the visual world (e.g., size, shape, or movement of objects) are called iconic gestures (McNeill, 1992). Speech and gesture are both semantically and temporally related; however, the onset of iconic gestures often proceed their semantic affiliates in speech (Fritz, Kita, Littlemore, & Krott, 2021; Morrel-Samuels & Krauss, 1992; ter Bekke, Drijvers, & Holler, 2020). The lexical affiliate is the word(s) most closely related to the gesture meaning. For example, in the sentence, “He picked up the book,” paired with a lifting gesture, “picked up” would be considered the lexical affiliate. In a corpus of conversational data, it was found that on average, the start of the gesture movement occurred 672 msec before the lexical affiliates, and the start of the meaningful stroke of the gesture movement occurred 215 msec before the lexical affiliates (ter Bekke et al., 2020). To comprehend the speech-gesture signal, listeners must integrate temporal and semantic features of speech and gesture during multimodal language processing. Many studies have used eye-tracking to examine spoken language processing as the speech signal unfolds in real time. However, the study of multimodal language processing has received much less attention. Using an adapted visual world paradigm, we examine how listeners use information from gesture to resolve temporary referential ambiguity in speech. Critically, we also examine whether this process is disrupted in individuals with moderate-severe traumatic brain injury (TBI), advancing our understanding of the effects of cognitive-communication impairment on speech-gesture integration in rich multimodal communication contexts.

### 1.1. Language processing in a visual world

The visual world paradigm (Tanenhaus, Spivey-Knowlton, Eberhard, & Sedivy, 1995) has been used to identify how and when language processing interacts with visual context by examining gaze to language-relevant entities in the visual world during the production and perception of language. When perceiving individual words, the listener’s eye fixations reveal partial activation of semantically and phonologically related competitors. For example, upon hearing both the words “lock” and “log”, participants show increased fixations to a picture of a key due to activation of semantic information associated with both the spoken word and its phonological competitors (Yee & Sedivy, 2006).

In addition to conceptual and phonological knowledge about words driving visual attention at the individual word level, words also occur within a larger linguistic context which can constrain or disambiguate meaning. For example, Altmann and Kamide (1999) measured fixations to objects in a scene while listening to sentences in which the verb constrained the candidate meanings for the upcoming object referent (e.g., “The boy will eat the cake,” where the cake was the only edible object in the scene) and sentences in which the verb did not constrain the referent (e.g., “The boy will move the cake”). They found that participants made anticipatory eye movements toward cake after hearing the verb “eat” more so than after hearing the verb “move.” These anticipatory effects also occur in the context of more ecologically valid real-world scenes and can be guided by contextually-relevant information in a scene in the absence of the target item (e.g., fixations to a table upon hearing “eat”) (Coco, Keller, & Malcolm, 2016).

During online sentence processing, listeners’ visual attention is influenced by both semantic and syntactic information conveyed by the words they hear, with fixations guided to candidate referents continuously and based on partial phonetic information (Dahan & Tanenhaus, 2004). Further, listeners use visual context to guide comprehension in the face of ambiguity in linguistic meaning (Ryskin, Qi, Duff, & Brown-Schmidt, 2017; Snedeker & Trueswell, 2004; Spivey, Tanenhaus, Eberhard, & Sedivy, 2002). For example, in sentences with a temporarily ambiguous prepositional phrase (e.g., “Put the apple on the towel in the box”), participants use the visual context to correctly disambiguate whether the phrase “on the towel” was intended to modify the noun apple (e.g., the apple that is on the towel) or to specify the goal of the verb *put* (e.g., where to put the apple, as in “Put the apple on the towel. Then move it to the box; see Spivey et al., 2002). The resolution of such syntactic ambiguities can be guided by both visual saliency of objects in the scene (e.g., color, intensity) and linguistic saliency (e.g., phrasing with intonational breaks) (Coco & Keller, 2015). Thus, language processing is contextual and requires the integration of both visual and linguistic input.

### 1.2. Contributions of gesture to language processing

Collectively, studies using the visual world paradigm provide several insights into the phonological, syntactic, and semantic constraints that influence on-line language processing and the ways language interacts with the visual world. However, all these studies used auditory-only language stimuli. Of note, recently, the visual world paradigm has been extended to manual languages in which the visual modality contains both linguistic (e.g., signs) and non-linguistic information (e.g., pictured objects). Both adult and child users of American Sign Language show evidence of semantic prediction, making anticipatory fixations to the target referent when the verb constrained the sentence-final noun (Lieberman & Borovsky, 2020; Lieberman, Borovsky, & Mayberry, 2018), and adult users of German Sign Language show phonological priming effects for sign pairs sharing hand shape and movement (Wienholz, Nuhbalaoglu, Steinbach, Herrmann, & Mani, 2021). Thus, addressees integrate visual context with linguistic information during language processing, even within a single visual modality. The current study examines multimodal language processing in which the signal contains both verbal and visual language in speech and gesture, respectively. Gesture is an integral component of language, with existing theories of gesture positing that gesture and spoken language share one common conceptual origin (McNeill, 1992, 2005, 2013; McNeill & Duncan, 2000) or are two closely integrated systems that interact during production (de Ruiter, 2000; Kita & Özyürek, 2003). Gestures provide temporal (Fritz et al., 2021; Morrel-Samuels & Krauss, 1992; ter Bekke et al., 2020) and semantic (McNeill, 1992) overlap with speech and can also constrain sentence meaning. For example, gestures can be used to disambiguate homophones (Holle & Gunter, 2007; Obermeier, Holle, & Gunter, 2011, 2012) and pronoun referents (Goodrich Smith & Hudson Kam, 2012, 2015). However, little is known about how gesture interacts with spoken language and visual context during language processing.

There has been increasing interest in expanding the visual world paradigm to study the interaction of gesture with visual context. Most of this early work has focused on the influence of iconic gestures on language processing although there is evidence that beat gestures (i.e., rhythmic movements) may also direct attention by emphasizing or stressing contrastive information (Morett, Fraundorf, & McPartland, 2021). Iconic gestures, in particular, play a role in predictive language processing due both to their semantic relatedness with the speech signal and tendency to occur before their lexical affiliates in speech (Hu, 2020). Saryazdi and Chambers (2022) examined how iconic features of grasping gestures reflecting the size and shape of object referents directed attention in a visual scene. For example, hearing the sentence “Pick up the candy” paired with a small pinching gesture on the word “Pick” diverts eye gaze to the target candy and away from the phonological competitor *candle*. This effect was moderated by object size, with a larger effect observed when the gesture differentiated a small target from a larger competitor in the visual scene.

**Fig. 1 –:**
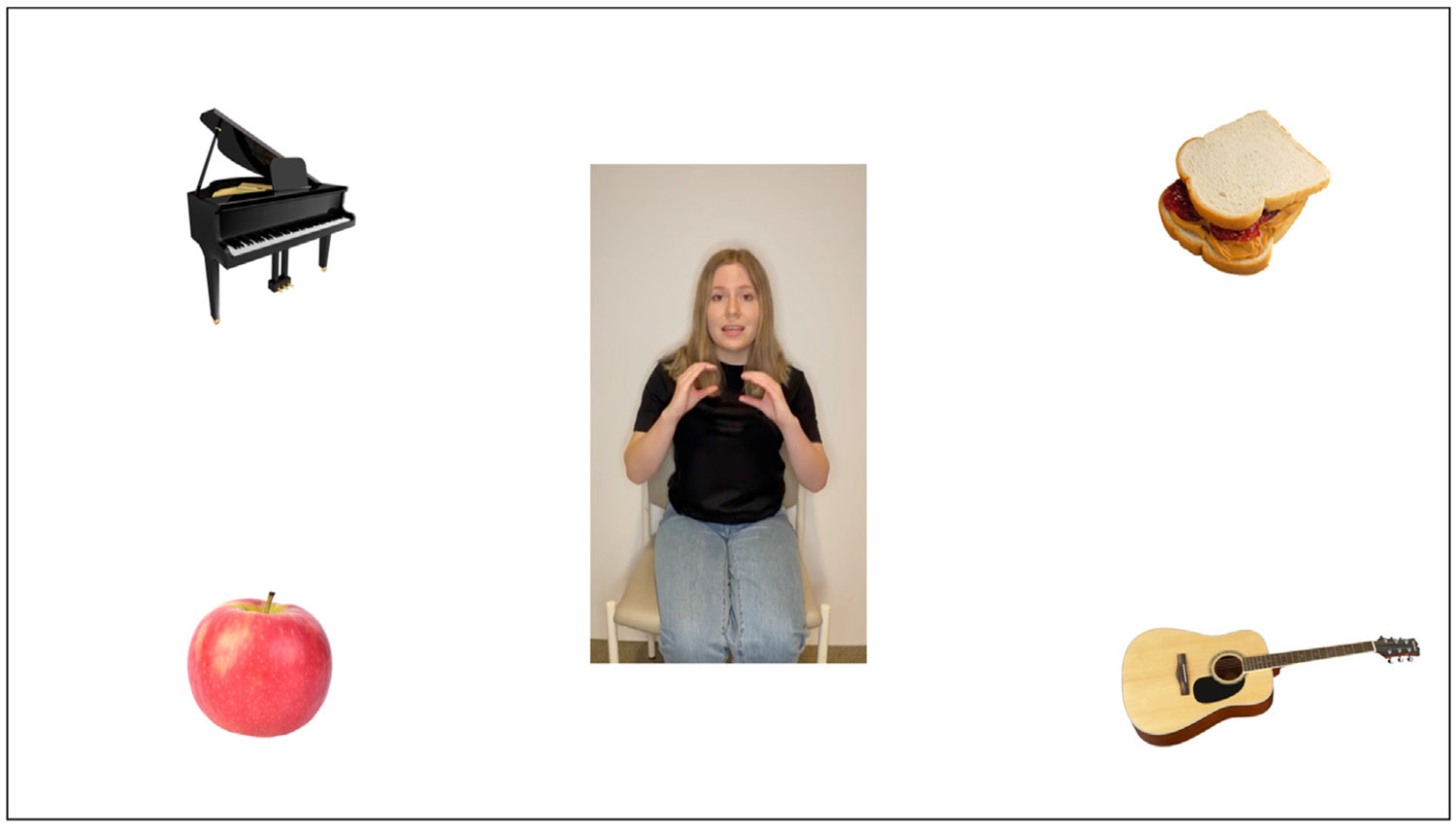
Example Trial in the Gesture Movement Condition. *Note.* Participants viewed a video of a speaker saying, “The girl will eat the very good sandwich,” while producing a *sandwich-holding* gesture on the verb, “will eat.” In this example, the target is *sandwich*, the semantic competitor is *apple*, and the distractor items are *piano* and *guitar*.

In a recent study, our group extended this line of work, using an adapted visual world paradigm, to examine how listeners use meaningful information provided uniquely in the gesture modality to resolve temporary ambiguity in speech (Clough, Duff, & Brown-Schmidt, 2023). Healthy young adults watched videos of a speaker producing subject-verb-object sentences in English (e.g., “The girl will eat the very good sandwich”) during which the speaker either produced a meaningful *sandwich-holding* gesture or a meaningless grooming movement (e.g., arm scratch) on the verb phase “will eat.” Each trial contained pictures of the target item (*sandwich*), a semantic competitor related to the verb (*apple*), and two distractor items (e.g., *piano, guitar*) (see Fig. 1 below). We found that participants were more likely to fixate the target item before hearing the referent in speech when the speaker produced a meaningful gesture on the verb. Gesture continued to have a facilitative effect on predictive target fixations in the presence of noise-degraded speech and when the speaker was wearing a surgical mask. Thus, the visual world paradigm has great utility for examining influences of multimodal language cues alongside speech in a visual context.

### 1.3. Disruptions to language processing in clinical populations

Another important application of the visual world paradigm is to study language processing in clinical and neurodiverse populations. This has the potential both to improve understanding about the communicative abilities of these populations and yield insights into the neural and cognitive resources that support language processing. For example, although they did not include gesture, Yee, Blumstein, and Sedivy (2008) leveraged the unique neural profile of individuals with Broca’s and Wernicke’s aphasia who have lesions to the anterior and posterior language regions, respectively, to examine the neural underpinnings of lexical processing. Participants listened to words spoken in isolation (e.g., “hammer”) while viewing an array of four pictures, one of which depicted a semantic competitor (e.g., *nail*) or a phonological competitor (e.g., *hammock*). Although participants with both Broca’s and Wernicke’s aphasia showed increased fixations to a semantic competitor of the target (e.g., looking at *nail* when they hear “hammer”), demonstrating lexical coactivation in line with healthy young and age-matched adults, they showed aberrant patterns of lexical activation when the competitor shared phonological onsets with the target. Whereas participants with Broca’s aphasia did not show significant increases in fixations to phonological competitors, participants with Wernicke’s aphasia showed stronger phonological competitor effects than their healthy age-matched comparisons.

Studying on-line language processing in individuals with bilateral hippocampal lesions and amnesia has also informed our understanding of the role of the hippocampus and memory in everyday language use. Although patients with amnesia show intact patterns of fixations to target items in a visual scene based on relatively preserved semantic and syntactic knowledge (Brown-Schmidt et al., 2021; Ryskin, Qi, Covington, Duff, & Brown-Schmidt, 2018), they show impairments in their abilities to link information across short sentences and discourse history to resolve linguistic ambiguity (Covington, Kurczek, Duff, & Brown-Schmidt, 2020; Kurczek, Brown-Schmidt, & Duff, 2013; Rubin, Brown-Schmidt, Duff, Tranel, & Cohen, 2011). Studies of on-line multimodal language processing in clinical populations are limited but ripe for investigation. It is possible that the relative weight of speech and gesture cues differs for individuals with cognitive or communication disorders. In face-to-face conversation, participants with aphasia were more likely to fixate on their communication partner’s gestures than were healthy comparison participants (Preisig et al., 2018), indicating that listeners may depend more heavily on gesture for comprehension in the case of language impairment. However, multimodal language processing may also tax cognitive resources. Using eye-tracking, Silverman, Bennetto, Campana, and Tanenhaus (2010) showed that although iconic gestures facilitated comprehension in neurotypical participants, it hindered comprehension in participants with high functioning autism, who showed patterns of impaired speechgesture integration. In the current study, we examine whether on-line speech gesture integration is disrupted in adults with moderate-severe TBI.

### 1.4. Speech-gesture integration in adults with traumatic brain injury

TBI is a disorder of brain connectivity that results in wide-spread damage to white matter tracts throughout the brain (Hayes, Bigler, & Verfaellie, 2016). The pattern of cognitive deficits is heterogeneous (Covington & Duff, 2021), and people with TBI can have disruptions to a variety of cognitive domains including memory (Bigler et al., 1996; Palacios et al., 2013; Rigon, Klooster, Crooks, & Duff, 2019, 2020; Velikonja et al., 2023), attention and processing speed, (Dockree et al., 2004; Ponsford et al., 2023; VanSolkema, McCann, Barker-Collo, & Foster, 2020), executive functioning (Jeffay et al., 2023; B. C. McDonald, Flashman, & Saykin, 2002), social cognition (Bibby & McDonald, 2005; S. McDonald & Flanagan, 2004; Turkstra, Norman, Mutlu, & Duff, 2018), and communication (Dahlberg et al., 2006; Togher et al., 2023). Individuals with TBI can present with deficits in multisensory processing and integration (Campbell et al., 2021; Kerley et al., 2020; S. McDonald, Dalton, Rushby, & Landin-Romero, 2019). However, the effects of these deficits on language processing are understudied. Successful communication requires integration of perceptual, emotional, and situational cues with shared world knowledge (MacDonald, 2017). Disruptions in the ability to process and integrate multiple cues may adversely affect social participation and may underlie the well-documented communicative impairments in TBI, such as impaired perception of irony (Martin & McDonald, 2005) and sarcasm (Channon, Pellijeff, & Rule, 2005).

Few studies have examined whether TBI impairs speech-gesture integration. One study examined gesture comprehension in isolation by asking participants to watch silent movies with gestures and found that participants with TBI were successful at interpreting both simple and complex communication acts (e.g., gesturing for someone to take a seat) but were impaired at interpreting gestures communicating deceit and irony (Bara, Cutica, & Tirassa, 2001). Another study found that although gestures combined with speech improved comprehension of indirect requests, participants with TBI were still impaired at interpreting indirect requests relative to noninjured comparison participants (Evans & Hux, 2011).

In a previous study by our group (Clough, Padilla, Brown-Schmidt, & Duff, 2023), we tested for behavioral evidence that participants with TBI integrated unique information provided only in gesture (e.g., a speaker saying, “He searched for a new recipe,” while producing a *typing* gesture) in their narrative retellings of stories. We found that participants with TBI did not significantly differ from non-injured peers in their probability of reporting information from gesture in their retellings (e.g., saying, “He searched for a new recipe *online*”) despite having poorer recall for stories overall. Although the literature is limited, collectively these studies provide evidence that gesture does influence language comprehension in TBI. However, to date, no studies have examined the on-line processing of speech and gesture in real time in TBI. It is possible that eye-tracking may more sensitively reveal delays or reductions in speech-gesture integration that are not always apparent in the downstream behavioral responses of adults with TBI. Using eye-tracking to examine multimodal processing in TBI not only has the potential to improve sensitivity of communication deficits in TBI but also could inform new treatment targets and improve our understanding of communicative abilities after brain injury.

### 1.5. Current study

We examined on-line speech-gesture integration in adults with and without moderate-severe TBI, using the same adapted visual world paradigm described above (Clough, Duff, & Brown-Schmidt, 2023). There is no overlap in the participant samples between the two studies. In addition, we used the same dataset in our methodological paper, Cho, Brown-Schmidt, Clough, and Duff (2024), as in this paper. The methodological paper presents a novel model specification for comparing group differences in trends across time within a trial and across a series of trials (over the course of an experiment) using generalized additive mixed models. However, the statistical models in the current paper are different from those in Cho et al. (2024). We used the dataset solely for the purpose of demonstrating the proposed statistical models in Cho et al. (2024).

Hypotheses and analysis plans for the current study were preregistered (https://osf.io/uyqv6), and data and analysis scripts are available on the OSF project: https://osf.io/6ga8c/

In the preregistration, we report how we determined our sample size, all data exclusions, all inclusion/exclusion criteria, whether inclusion/exclusion criteria were established prior to data analysis, all manipulations, and all measures in the study. As in healthy young adults, we predicted that participants would be more likely to look at the target picture (e.g., *sandwich*) during the critical analysis window (the period of ambiguity in the sentence when the speech stream had not yet uniquely identified the upcoming referent) when the speaker produced a meaningful gesture compared to a meaningless grooming movement. We expected a main effect of group, such that participants with TBI would be less likely to fixate on the target item during the critical analysis window than non-injured participants. Our primary hypothesis was that individuals in the TBI group would demonstrate impaired speech-gesture integration. Thus, we also predicted a group-by-movement type interaction such that the effect of gesture on fixations to the target would be smaller in the TBI group relative to the non-injured group. As this is the first study of on-line speech-gesture integration in individuals with TBI, the primary aim was to identify whether an impairment in speech-gesture integration is present at the group level. An important follow-up question is what individual differences in cognitive or neural profiles might drive these group differences. There are a growing number of studies linking co-speech gesture processing to working memory in neurologically healthy adults (Aldugom, Fenn, & Cook, 2020; Wu & Coulson, 2014, 2015; Ö zer & Göksun, 2020b). Therefore, as a first step in an exploratory analysis, we examine whether differences in working memory predict sensitivity to gesture in the TBI group.

Although we predicted that participants with TBI would show reduced speech-gesture integration overall during rapid language processing, we did not have a specific hypothesis about how their visual attention to the visual scene would differ from their NC peers. To explore these differences, we conducted a pre-registered supplementary analysis to determine which competing locations in the visual scene captured participants’ attention across groups and movement conditions (see IRTree analysis below). These exploratory analyses build a foundation for future confirmatory studies to examine the mechanisms underlying multimodal integration and processing difficulties in TBI.

## 2. Methods

### 2.1. Participants

Participants were 45 adults with moderate-severe TBI and 45 non-injured comparison (NC) participants. Due to an equipment failure that led to >50% data loss, one NC participant was excluded from analysis, as per our preregistration protocol. Thus, the final sample was 45 participants with TBI and 44 NC participants. The two groups were matched on sex, age, and education (Table 1).

Participants were recruited from the Vanderbilt Brain Injury Patient Registry (Duff et al., 2022). All participants with TBI sustained their injuries in adulthood and were in the chronic stage of recovery, at least 6 months post injury. Participants were classified as moderate-severe by the Mayo Classification System (Malec et al., 2007) and met at least one of the following criteria: (1) Glasgow Coma Scale (GCS) < 13 within 24 h of acute care admission; (2) positive neuroimaging findings (acute CT findings or lesions visible on a chronic MRI); (3) loss of consciousness (LOC) > 30 min; or (4) post-traumatic amnesia PTA >24 h. See Table 2 for a summary of individual injury demographics.

To characterize the cognitive profiles of the sample, we report neuropsychological test results for a subset of the participants (n_TBI_ = 42, n_NC_ = 26) who completed the NIH Toolbox Cognition Battery (Weintraub et al., 2013) on an iPad during a separate session through their participation in the Vanderbilt Brain Injury Registry. Subtests include measures of attention, working memory, episodic memory, executive function, language, and processing speed, as well as composite scores for fluid, crystallized, and total cognition. The NIH Toolbox Cognitive Battery has established psychometric properties (Heaton et al., 2014; Carlozzi et al., 2017; Tulsky et al., 2017) and is recommended for use in TBI research (e.g., NIH Common Data Elements). Table 3 reports the age-corrected standard scores for all subtests and composite scores by group, and individual scores are available on the OSF project. The TBI group scored significantly lower on all subtests and composites relative to the NC group.

### 2.2. Stimuli

Participants watched videos of a speaker producing subject-verb-object sentences (e.g., “*The girl will eat the very good sandwich*.”). During onset of the verb phrase (e.g., “*will eat*”), the speaker sometimes produced a meaningful iconic gesture that could be used to identify the upcoming target referent (e.g., a *sandwich-holding* gesture). On other trials, the speaker produced a meaningless grooming (self-touch) movement (e.g., an arm scratch movement). The movements were triphasic (McNeill, 1992), consisting of a preparation phase in which the speaker lifted their hands from resting position, the movement stroke (i.e., either the meaningful portion of the gesture or the act of performing the grooming movement), and a retraction phase in which the speaker returned their hands to a resting position. An intonational break (*M* = 427 msec, *SD* = 149 msec) between the verb and the adjective phrase was inserted to increase the length of the anticipatory window between the movement and target word. There were 80 unique stimulus sentences (See OSF project for stimulus list). Two versions were recorded for each sentence, one with a meaningful gesture and another version with a meaningless grooming movement, by each of four speakers of North American English (2 male, 2 female). Thus, each of the 80 sentences was recorded eight times (2 conditions x 4 speakers), creating a total of 640 stimulus videos with unique target, movement, and speaker combinations. These 640 items were put into a single randomized order and then divided into 8 blocks of 80 trials each. From the 8 blocks, we used a Latin square sampling design to create 8 stimulus lists of all possible consecutive orders of 3 blocks (e.g., blocks 1–3, blocks 2–4, blocks 3–5, etc.). Participants were randomly assigned to one of these lists of 240 trials, so that all participants viewed a variety of target items and speakers and a subset of all possible stimulus items. The stimulus videos were placed in a visual scene. On each trial, participants viewed the video in the middle of the screen and saw four picture objects, one in each of the four corners. These objects consisted of the target item (e.g., *sandwich*), a semantically related competitor item (e.g., *apple*), and two distractor items (e.g., *piano* and *guitar*). The location of the target and competitor items were randomized across trials. See Fig. 1 for an example trial.

### 2.3. Procedure

Participants were seated at a computer with a desktop-mounted Eye-link 1000 eye-tracker (SR Research) with their head stabilized in a padded chin rest. Participants were fitted with Bluetooth cordless and noise-canceling headphones and listened to a sample audio file to ensure audibility. All participants self-reported the audio sample was sufficiently loud. Participants were given the following instructions: “On each trial, you will see one video and four pictures. In the video, a speaker will talk about a girl. The speaker will tell you what tasks the girl needs to do. Your job is to click the object the girl needs to perform the task.” Participants then completed two practice trials to ensure they understood the directions. On a given trial, the objects appeared on the screen immediately. The video appeared after a 1-s delay. The video disappeared after it finished playing, and the pictures remained on the screen until the participant clicked one. A drift-check occurred every five trials. If the drift-check failed, the eyetracker was re-calibrated. The experiment took approximately 45 min to complete. Participants were offered breaks every 80 trials (i.e., two breaks at 15-min intervals).

### 2.4. Analysis

Accuracy in clicking the picture corresponding to the target word was >97.5% for all participants. All participants had minimal data loss, with fixations recorded to one of the five regions of interest (corresponding to the video or 4 object locations) at high proportions; across all 10 msec bins of tracking data across all trials, the proportion of bins with fixations to one of the five regions of interest ranged from .87 to 1.0 in the NC group (*M* = .97, *SD* = .03) and .88 to 1.0 in the TBI group (*M* = .97, *SD* = .03).

**Fig. 2 –:**
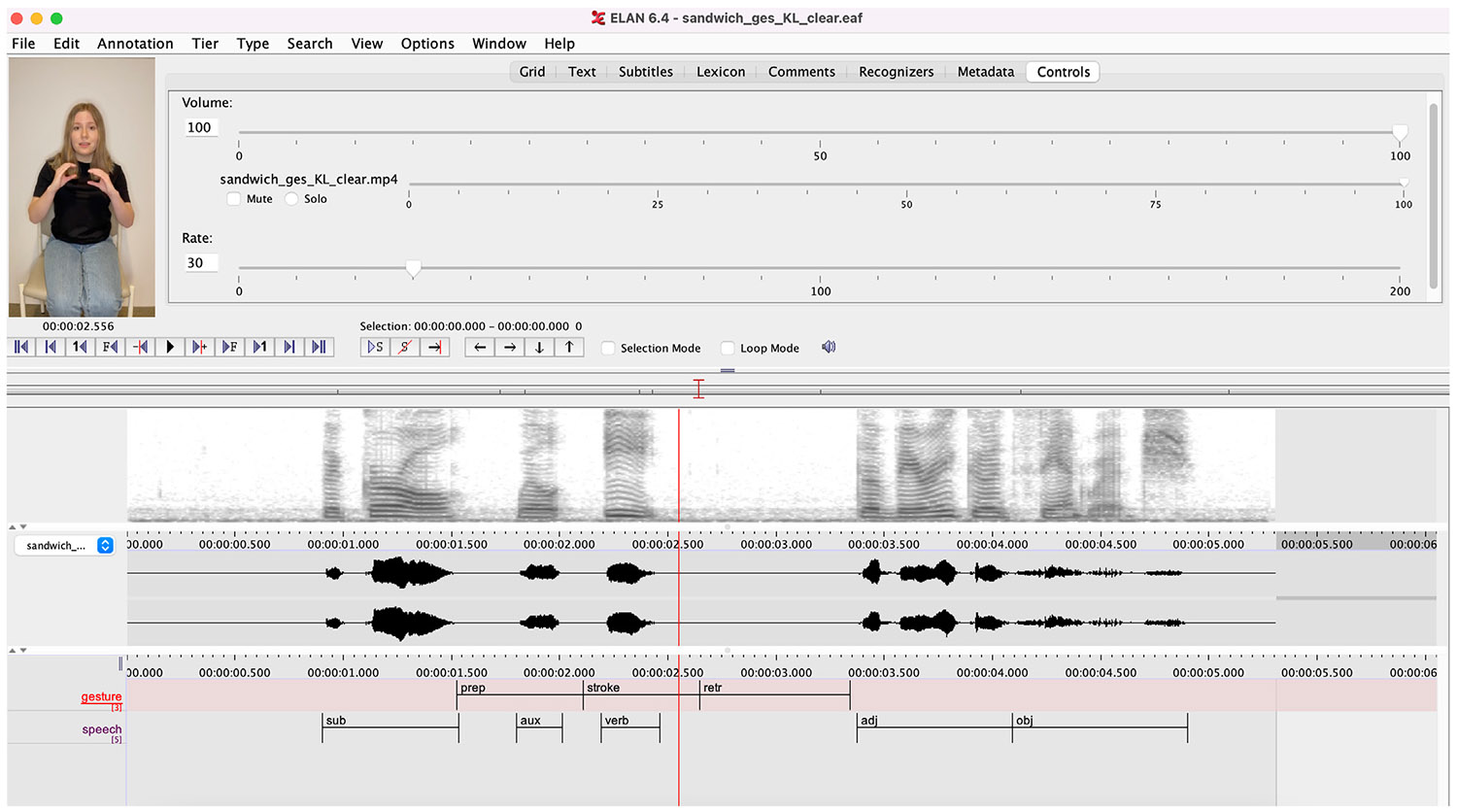
Example Timestamp Coding for Stimulus Items. *Note.* Gesture and grooming movements were coded as triphasic, where each movement consisted of a preparation phase *(prep)* that began when the speaker’s hands lifted from their lap, a stroke phase *(stroke)* that began when the speaker’s hands paused at maximal height and continued through the duration of the gesture or grooming form, and a retraction phase *(retr)* that began when the speaker began to drop their hands to a resting position. The auditory signal was coded into subject (*sub;* e.g., “The girl”), auxiliary verb (*aux;* e.g., “will”), main verb (*verb;* e.g., “eat”), adjective phrase (*adj;* “the very good”), and object components (*obj;* e.g., “sandwich”).

The dependent measure was binary fixations to the target object in each of a series of 10 msec bins across all trials within a participant. For the primary analysis, we used *dynamic generalized linear mixed models* (dGLMM), an extension of autoregressive mixed-effect models (Cho, Brown-Schmidt, & Lee, 2018) to predict the probability of fixations to the target (1) or not (0) across the entire critical analysis window. The analysis window is the same size for all trials in fitting dGLMM. The analysis window began 180 msec after the onset of the speakers’ movement stroke for both the gesture and grooming movement conditions, coded uniquely for each stimulus video using ELAN version 6.4 (Fig. 2) and ended at the average onset of the target object word produced in speech, 2700 msec later. Thus, all trials were aligned at the beginning of the movement stroke, and we examined fixations to the target object collapsed across the critical window between movement stroke and the average onset of spoken target referent. This analysis window was offset by 180 msec due to the time needed to launch an eye-movement (Boff, Kaufman, & Thomas, 1986), minus a 20 msec baseline to model the first-order autocorrelation (AR(1)).

Fixed effects included participant group (dummy coded with NC as reference level; NC = 0, TBI = 1), movement type (effects coded; grooming movement = −.5, gesture = .5), trial number (i.e., how far along participants were in the experiment, mean centered and scaled for estimation stability) and their interactions. Because the NC group was dummy coded as the reference level, all main effects in the model (e.g., the movement type condition effect) are interpreted as the simple effect for non-injured participants (the reference level for the participant group variable). To test whether effects differed between the NC and TBI groups, we looked for significant interactions between participant group and movement type/trial number. Significant interactions with participant group indicate that the magnitude (and/or direction) of an effect significantly differs between the NC and TBI groups. To probe and interpret any significant interactions, we reverse-dummy coded the model, setting the TBI group as the reference level to determine the simple effects for the TBI group as well. The effect of movement type was effects coded so that effects of Group and Trial Number could be interpreted as main effects, rather than simple effects at one level of the Movement type variable. Although every trial that participants saw in the experiment contained a unique stimulus video (i.e., a unique combination of sentence, movement, and speaker), target items and movement types were repeated across trials (stimulus items available in OSF project). Including trial number as a fixed effect allows us to determine differences between groups in learning trends across the course of the experiment. We also included fixed effect covariates for AR(1) (effects coded; no previous target fixation = −.5, previous target fixation = .5), and time (mean-centered and scaled for estimation stability) to account for moment-to-moment dependencies in the data in participants’ fixation locations. Models were conducted using the *lme4* (Version 1.1–35.2; Bates, Mächler, Bolker, & Walker, 2015), *Matrix* (Version 1.6–5; Bates, Maechler, & Jagan, 2024), and optimx (Version 2023–10.21; Nash, 2014; Nash & Varadhan, 2011) packages in R version 4.3.3 (R Core Team, 2024), and the random-effect structure was determined using the *Buildmer* package in R (Version 2.11; Voeten, 2023). The maximal model included random slopes for movement type, trial number, time, and AR(1) by participant and a random slope for items. The selected model included a random slope for AR(1) and Condition by participant and a random intercept for items.

Following this analysis, we conducted a post hoc exploratory analysis to examine whether differences in the List Sorting Working Memory subtest from the NIH Toolbox Cognitive Battery interacted with movement condition effects. Because we only had working memory scores for *n* = 42 participants with TBI and *n* = 26 NC participants, we were underpowered to detect a three-way interaction between group, movement condition, and working memory. Thus, we examined results for the TBI group only and, upon reviewer request, report a separate similar model for the NC group. We used a dGLMM model with an nlminbwrap optimizer to predict binary target fixations (1 = fixations to target; 0 = fixations anywhere else) as a function of movement condition, trial number, working memory scores (mean centered and scaled), and their interactions, with covariates for AR(1) and Time with random slopes for AR(1) and movement condition by participant and a random intercept for items.

A further exploratory supplementary analysis examined the data using a dynamic tree-based item response model (IRTree; Cho, Brown-Schmidt, Boeck, & Shen, 2020), which models the data in polytomous form at three nodes. Whereas the dynamic GLMM above examines fixations to the target or not, the dynamic IRTree model considers fixations to other competing locations on the screen which allowed us to examine where participants are looking when they are not looking at the target item and whether fixation behavior across these locations differs by group. The data were coded into the following categories: target fixations, competitor fixations, fixations to the other two distractor pictures, and fixations to the video. Then, at each node the data were re-coded into binary form as follows: Node 1 distinguishes fixations to the video (0) from the other three categories (1). Node 2 distinguishes fixations to verb-relevant pictures (e.g., target or competitor = 1) from distractor pictures and everything else (0). Node 3 distinguishes fixations to target (1) from the competitor object (0). Each node in the model included the same fixed effects as in the dynamic GLMM model as described above. We compared model fit of three nested models: Model 1 included random intercepts only for participant and item at each node. Model 2 included a random slope for the AR(1) effect by node for participant and a random intercept for item. Model 3 included a random slope for the AR(1) effect and movement type for participant and a random intercept for item. We used the anova() function in R to compute a likelihood ratio test to determine whether the more complex models improved data fit over simpler models, while accounting for model complexity. We found that Model 3 was a significantly better fit to the data and was selected as the final model, reported below. Scripts for the binary and IRTree analyses are available on the OSF project.

## 3. Results

### 3.1. Analysis of binary fixations to a target item

**Fig. 3 –:**
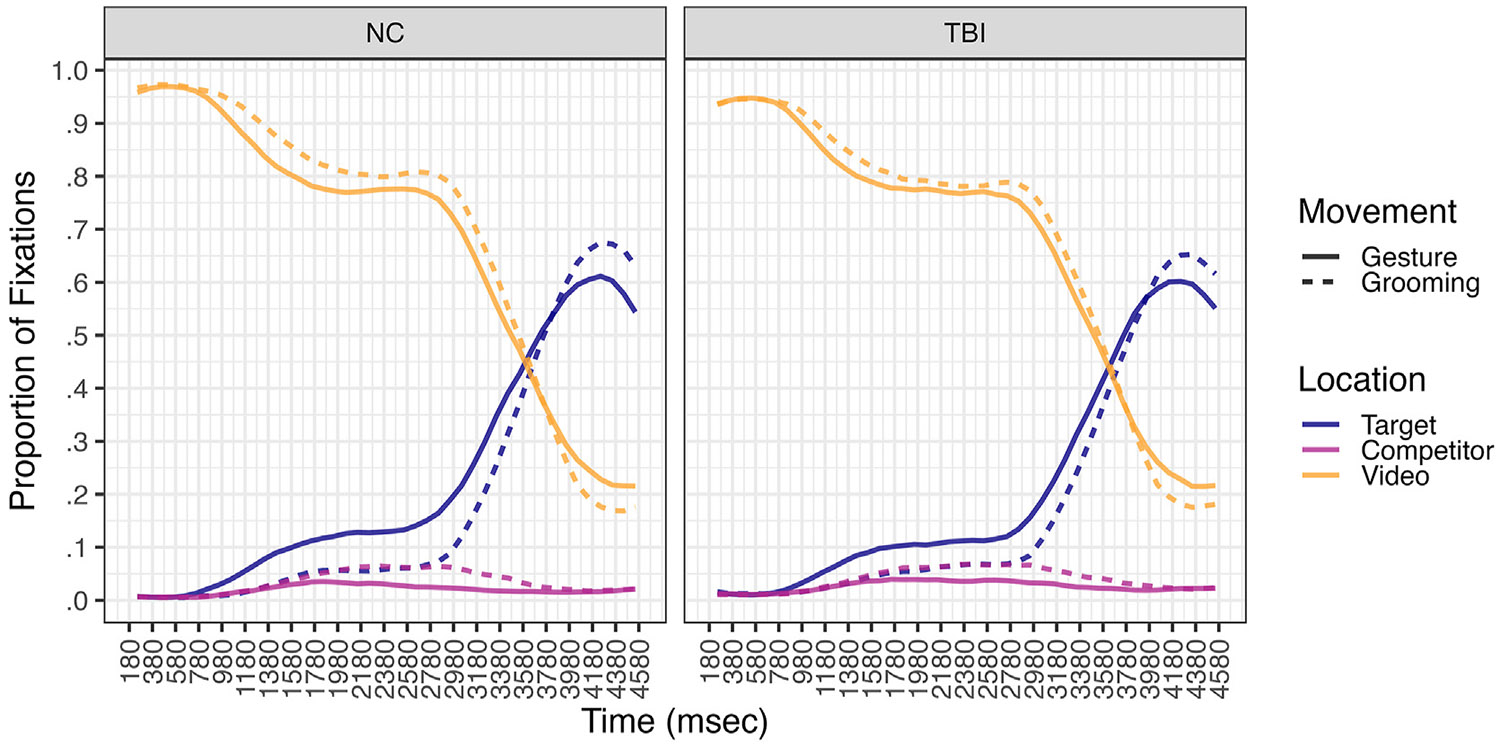
Average Proportion of Fixations to Target, Competitor, and Video Across All Trials by Group. *Note.* Time on the x-axis starts at 180 msec after gesture or grooming movement stroke and ends at the average response time of participants’ picture click.

**Fig. 4 –:**
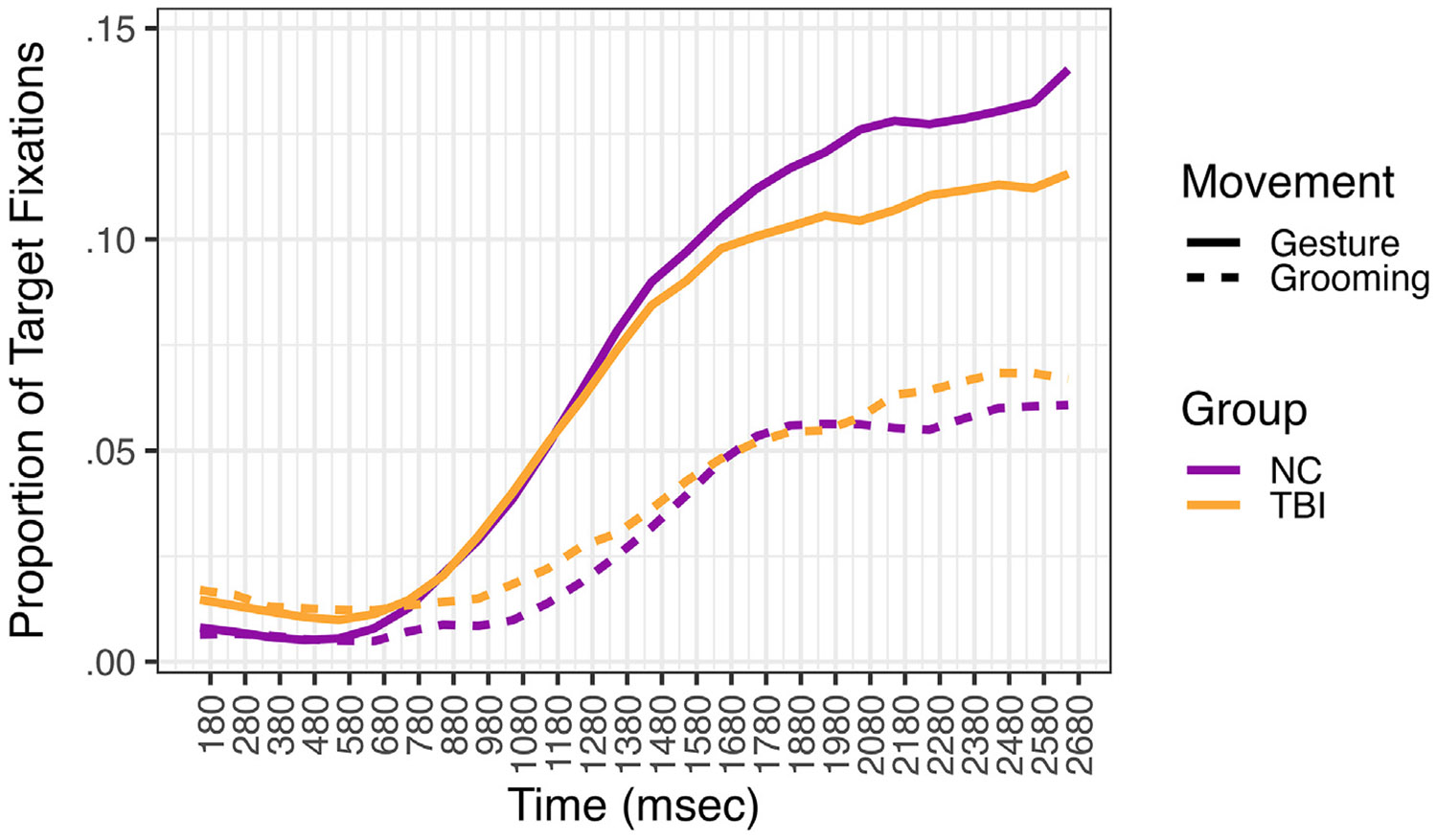
Proportion of Fixations to Target Item by Participant Group and Movement Type. *Note.* The critical analysis window began 180 msec after the onset of the movement stroke produced by the speaker in gesture and ended at the average onset of the target referent produced by the speaker in speech.

We present the overall timecourse of fixation proportions to the video, target item and competitor item locations averaged across all trials for the NC and TBI groups in Fig. 3. Proportion of target fixations by group and movement type during the critical analysis window are presented in Fig. 4, illustrating the effects reported below. Supplementary materials show the individual effects of movement type by participant in the NC (Fig. SI 1) and TBI (Fig. SI 2) groups. We modeled the probability of binary fixations to the target item as a function of participant group, movement type, trial number, and their interaction with covariates for Time and AR(1) across the entire critical analysis window between onset of movement stroke and average onset of the target item in speech.^1^Random slopes for Movement type and AR(1) by participant reflect individual differences in these effects. The *SD* of .29 for Movement type and 1.15 for AR(1) indicate non-ignorable variability across individuals. We visualize individual differences using predicted random effects in supplementary materials (Fig. SI 3). Results of the dynamic GLMM model with the NC group as the reference level are presented in Table 4. To interpret significant interactions with group, we reverse dummy coded the model setting the TBI group as the reference level. A table of these results are available in supplementary materials (SI Table 2).

There was a significant effect of movement type ($\hat{\beta }$ = .59, z = 8.94, *p* < .001); NC participants were 1.81 times more likely to fixate the target item during the critical analysis window when the speaker produced a meaningful gesture compared to a meaningless grooming movement. There was no significant effect of group ($\hat{\beta }$ = .15, z = 1.47, *p* = .14), indicating that participants with TBI did not significantly differ from NC participants in their probability of fixating the target item. A significant group*movement type interaction ($\hat{\beta }$ = −.17, z = −2.09, *p* = .04) indicated that the effect of movement type differed by group. To probe this interaction, we reversedummy coded the variables, setting the TBI group as the reference level. Although the effect of movement type was also significant for TBI participants ($\hat{\beta }$ = .42, z = 7.03, *p* < .001), it was attenuated in the TBI group. Whereas NC participants were 1.81 times more likely to fixate the target item when the speaker produced a gesture compared to grooming movement, participants with TBI were 1.52 times more likely to fixate the target item with gesture (see Fig. 4).

There was a significant effect of trial number on probability of fixations to the target ($\hat{\beta }$ = −.04, z = −2.54, *p* = .01); across both grooming and gesture trials, the probability of fixating the target item during the critical window decreased over the course of the experiment for non-injured participants. A lack of significant interaction between group and trial number ($\hat{\beta }$ = −.03, z = −1.18, *p* = .24) indicated that the magnitude of effect was not significantly different for participants with TBI. There was a significant interaction between movement type and trial number ($\hat{\beta }$ = .11, z = 3.77, *p* < .001), where the positive effect of gesture on the probability of target fixations increased across trials of the experiment. There was no threeway interaction between movement type, trial number, and group ($\hat{\beta }$ = −.04, z = −1.07, *p* = .29). The significant effect of time ($\hat{\beta }$ = .51, z = 32.42, *p* < .001) reflects the increasing probability of target fixations over time within a trial, and the significant effect of AR1 ($\hat{\beta }$ = 11.33, z = 90.90, *p* < .001) reflects the serial dependency from time-point to time-point in whether or not participants fixated the target at a given time point.

### 3.2. Analysis of dynamic tree-based item fixations

**Fig. 5 –:**
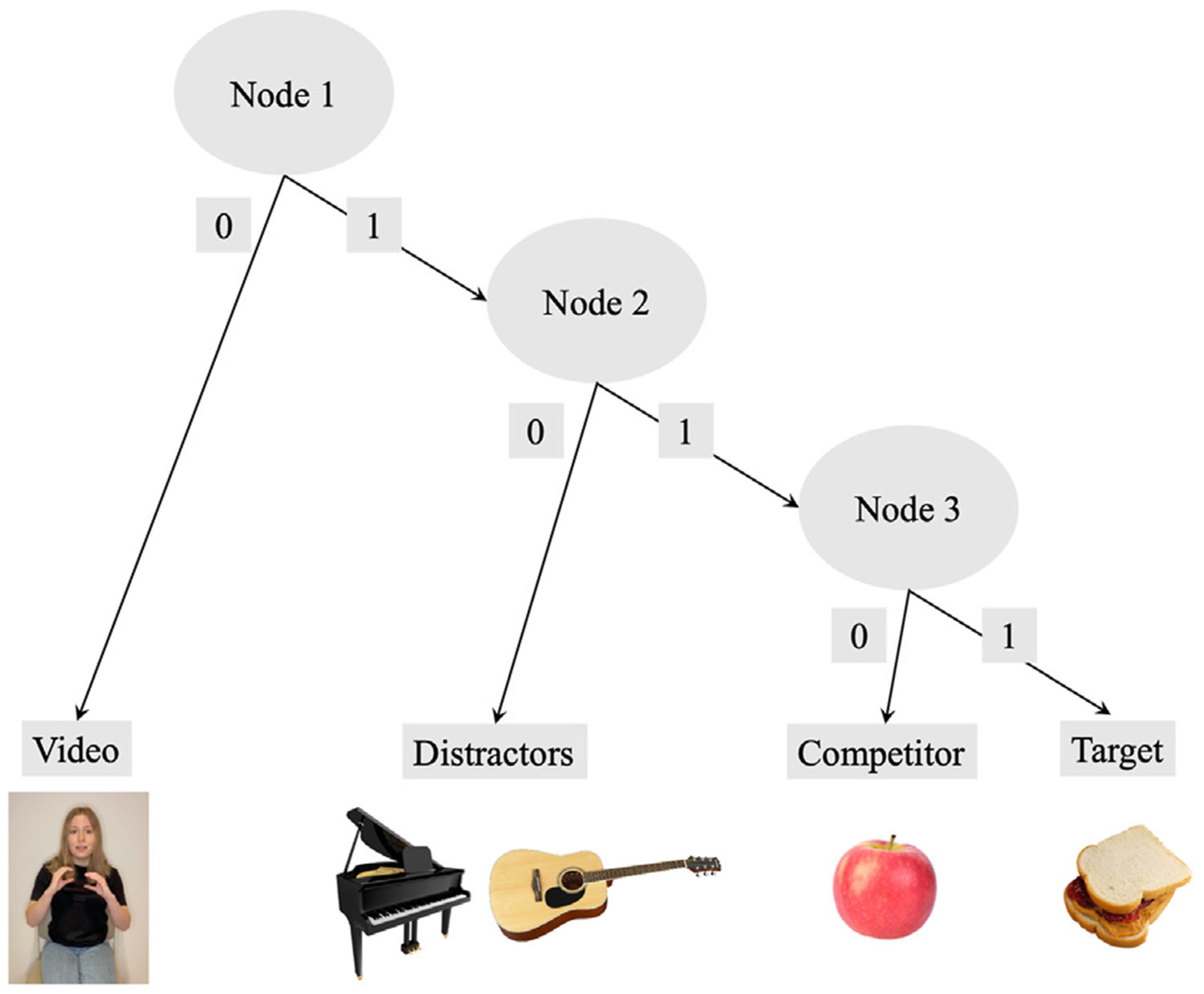
Tree diagram for dynamic IRTree model. Note. This four-category model had three nodes, each with two branches. Example fixation locations are shown for the sentence, “The girl will eat the very good sandwich.” Node 1 examines binary fixations to the Video compared to all pictured objects (Distractors + Competitor + Target). Node 2 examines binary fixations to Distractors compared to verb-relevant pictures (Competitor + Target). Node 3 examines binary fixations to Competitor compared to Target.

**Fig. 6 –:**
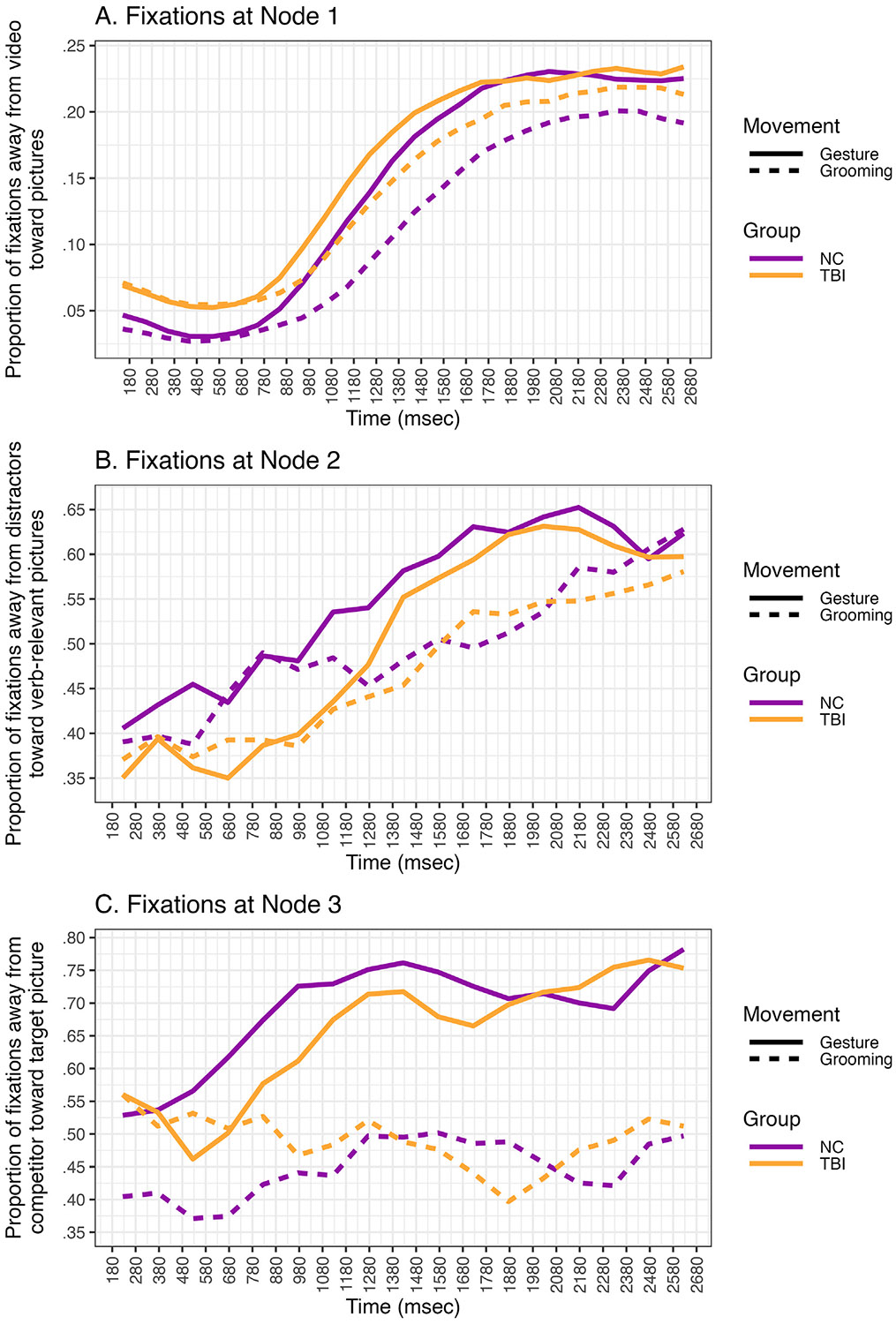
Proportion of fixations at each node of IRTree analysis by participant group and movement type.

The results from the binary model above examine the probability of fixating the target (1) over all other possible fixation locations (0). To supplement this analysis, we also present results from a dynamic IRTree model (Cho et al., 2020) which allows us to examine the probability of fixations to several competing locations on the screen. We tested effects of dependent variables (movement type, group, trial number) and covariates (time, AR(1)) at three nodes (Fig. 5). Node 1 examined the probability of fixating the pictures (1) compared to the video (0). Node 2 examined the probability of fixating the verb-relevant pictures (target or competitor; 1) compared to the distractor pictures (0); fixations to the video were treated as missing data at this node. Node 3 tested the probability of fixating the target (1) compared to the competitor (0); fixations to the video and distractor pictures were treated as missing data at this node. Results of the dynamic IRTree model are reported in Table 5 and visualized in Fig. 6. To interpret significant interactions with group, we reverse dummy coded the model setting the TBI group as the reference level. A table of these results are available in supplementary materials (SI Table 3).

#### 3.2.1. Node 1: fixations to pictures vs. video

Node 1 examines participants’ probability of fixating the pictured objects (1) compared to the video of the speaker (0) across the entire the critical ambiguous window (Fig. 6a). There was a significant effect of movement type in the Node 1 analysis ($\hat{\beta }$ = .23, *z* = 6.68, *p* < .001); NC participants were 1.26 times more likely to look away from the video and toward pictured objects when the speaker produced a meaningful gesture compared to a meaningless grooming movement. There was no significant effect of group in the Node 1 analysis ($\hat{\beta }$ = .15, z = 1.42, p = .16), indicating that participants with TBI did not significantly differ from NC participants in their probability of fixating the pictures vs. video. A significant group*movement type interaction ($\hat{\beta }$ = −.09, *z* = −1.98, *p* = .05) indicated that the effect of movement type in the Node 1 analysis differed by group. To probe this interaction, we reverse-dummy coded the group variable, setting the TBI group as the reference level. Although the effect of movement type was also significant for TBI participants (*β* = .14, *z* = 4.18, *p* < .001), the effect of gesture on diverting fixations away from video and toward pictures was smaller than in the TBI group; participants with TBI were 1.15 times more likely to look away from the video and toward pictured objects when the speaker produced a meaningful gesture compared to a meaningless grooming movement.

There was a significant effect of trial number on probability of fixations to pictured objects in the Node 1 analysis (*β* = −.001, *z* = −5.26, *p* < .001); across both grooming and gesture trials, the probability of looking toward pictures and away from the video decreased over the course of the experiment for non-injured participants. A significant interaction between group and trial number (*β* = −.001, *z* = −4.02, *p* < .001) indicated that this effect was significantly different for participants with TBI. Re-coding the group variable with the TBI group as the reference revealed that the probability of looking away from the video significantly increased across trials of the experiment for the TBI group as well (*β* = −.002, *z* = −11.51, *p* < .001), and that the magnitude of this effect was significantly larger in the TBI group than the NC group. There was a significant interaction between movement type and trial number (b = .001, *z* = 3.48, *p* < .001), where the positive effect of gesture on diverting attention away from the video and toward the pictures increased across trials of the experiment. There was no three-way interaction between movement type, trial number, and group in the Node 1 analysis (*β* = −.001, *z* = −1.41, *p* = .16), indicating that this across-trial learning to look toward pictures when the speaker produces a gesture was similar in the NC and TBI groups. The significant effect of time in the Node 1 analysis (*β* = .004, *z* = 41.44, *p* < .001) reflects the increasing probability of target fixations across time within a trial. The significant effect of AR1 in the node analysis (*β* = 10.66, *z* = 89.70, *p* < .001) reflects the serial dependency from time-point to time-point in whether or not participants fixated the pictures at a given time point.

#### 3.2.2. Node 2: fixations to verb-relevant pictures vs. distractor pictures

Node 2 examines participants’ probability of fixating the verb-relevant picture pair (e.g., the target or the competitor) (1) compared to the distractor pictures (0) across the entire critical ambiguous window (Fig. 6b). There was a significant effect of movement type in the Node 2 analysis (*β* = .27, *z* = 5.22, *p* < .001); When NC participants were looking at the picture stimuli, they were 1.32 times more likely to look at the target/competitor than the distractor pictures when the speaker produced a meaningful gesture compared to a meaningless grooming movement. There was no significant effect of group in the Node 2 analysis (*β* = −.16, *z* = −1.65, *p* = .10), indicating that participants with TBI did not significantly differ from NC participants in their probability of fixating the verb-relevant pair. A significant group*movement type interaction (*β* = −.19, *z* = −2.65, *p* = .008) indicated that the effect of movement type in the Node 2 analysis differed by group. To probe this interaction, we set the TBI group as the reference level. Whereas NC participants were more likely to look at the verb-relevant pictures when the speaker gestures, the effect on gesture on fixations to verb-relevant pictures was not significant in the TBI group (*β* = .09, *z* = 1.74, *p* = .08).

There was no significant effect of trial number on probability of fixations to verb-relevant pictures in the Node 2 analysis (*β* = .000, *z* = −.09, p = .93). The interaction between trial number and group (*β* = .000, *z* = −.02, p = .98) and the interaction between trial number and movement type in the Node 2 analysis were also both non-significant (*β* = .000, *z* = −.66, *p* = .51). There was no three-way interaction between movement type, trial number, and group in the Node 2 analysis (*β* = .001, *z* = 1.22, *p* = .22). The significant effect of time in the Node 2 analysis (*β* = .002, *z* = 6.19, *p* < .001) reflects the increasing probability of fixations to verb-relevant pictures over time within a trial, and the significant effect of AR1 in the Node 2 analysis (*β* = 10.11, *z* = 109.61, *p* < .001) reflects the serial dependency from time-point to time-point in whether or not participants fixated the verb-relevant pictures.

#### 3.2.3. Node 3: fixations to target vs. competitor

Node 3 examines participants’ probability of fixating the target (1) compared to the competitor (0) across the entire critical ambiguous window (Fig. 6c). There was a significant effect of movement type in the Node 3 analysis (*β* = .99, *z* = 8.32, *p* < .001); When NC participants were looking at the verb-relevant pictures, were 2.70 times more likely to look at the target than the competitor when the speaker produced a meaningful gesture compared to a meaningless grooming movement. There was no significant effect of group in the Node 3 analysis (*β* = −.14, *z* = −1.90, *p* = .06), indicating that participants with TBI did not significantly differ from NC participants in their probability of fixating the target vs. competitor. A lack of significant interaction between movement type and group (*β* = −.22, *z* = −1.40, *p* = .16) indicated that the magnitude of the effect of gesture on directing fixations to the target relative to competitor picture also did not significantly differ by group.

There was no significant effect of trial number on probability of fixations to the target picture in the Node 3 analysis (*β* = .001, *z* = 1.28, *p* = .20). The interaction between trial number and group was also not significant (*β* = .001, *z* = .93, *p* = .35). A significant interaction between trial number and movement type in the Node 3 analysis (*β* = .003, *z* = 2.57, *p* = .01) indicated that the beneficial effect of gesture on directing attention to the target picture increased across trials of the experiment. There was no three-way interaction between movement type, trial number, and group in the Node 3 analysis (*β* = −.001, *z* = −.76, *p* = .45). The effect of time in the Node 3 analysis was not significant (*β* = .001, *z* = 1.04, *p* = .30) indicating that the relative probability of fixating the target vs. competitor did not change over time within a trial. The significant effect of AR1 in the Node 3 analysis (*β* = 11.79, *z* = 73.01, *p* < .001) reflects the serial dependency from time-point to time-point in whether or not participants fixated the target picture at a given time point.

### 3.3. Exploratory analysis: cognitive predictors of speech-gesture integration

A growing number of studies have linked co-speech gesture processing to working memory in neurologically healthy adults (Aldugom et al., 2020; Wu & Coulson, 2014, 2015; Özer & Göksun, 2020b). Using data available from the NIH Toolbox Cognitive Battery, we conducted an exploratory analysis to examine the role of working memory (List Sorting subtest) in predicting target fixations across movement conditions for a subset of participants in the TBI group (42/45). We found no main effect of working memory on target fixations (*β* = −.04, *z* = −.24, *p* = .81). There was also no significant interaction between working memory and movement type (*β* = .14, *z* = 1.48, *p* = .14), suggesting no differentiation between participants across the range of working memory scores in the magnitude of the effect of gesture on target fixations. There was, however, a significant three-way interaction between movement type, trial number, and working memory scores (*β* = 0.12; *z* = 2.07; *p* = .04), suggesting that while participants with TBI who had higher working memory scores tended to show a stronger condition effect across trials of the experiment, those with lower working memory scores tended to show a decreased condition effect across trials. There were no significant interactions between working memory and movement condition in the smaller NC subgroup. Full results of the exploratory analysis are reported in supplementary materials (Supplementary Analysis 2).

## 4. Discussion

During language processing, listeners are exposed to communicative information from multiple modalities. In addition to the unfolding speech signal, language occurs in rich visual contexts, and speakers produce visible language in the form of gestures. Yet, few studies have examined the influence of gesture on language processing. We demonstrated that listeners use information from gesture to resolve temporary ambiguity in speech, making more anticipatory fixations to the target item when the speaker produces an iconic gesture on the verb. This replicates previous work by our group examining on-line speech gesture integration in undergraduate students (Clough, Duff, & Brown-Schmidt, 2023) and extends this finding to a larger and more age- and education-diverse sample of non-injured participants. The critical question addressed by the current study was whether traumatic brain injury disrupts on-line speech-gesture integration in an adapted visual world paradigm.

Both the NC and TBI groups used information from gesture to direct gaze towards target objects in the anticipatory period; however, non-injured participants did so to a greater extent than participants with TBI. This suggests that adults with TBI can integrate meaningful information from gesture during online language processing, but they may benefit less from gesture than non-injured peers. Our supplementary dynamic tree-based item analysis allowed us to disentangle where disruptions in the processing of multimodal language in a visual scene occurred in participants with TBI. In the NC group, we found that gesture increased the probability of fixations to relevant items in the scene across all three nodes: It facilitated fixations away from the video of the speaker and toward pictured items (Node 1), it facilitated fixations away from distractor pictures and toward verb-relevant pictures (Node 2), and it facilitated fixations away from the semantic competitor and toward the target picture (Node 3). Thus, gesture is effective at directing attention away from irrelevant information and toward relevant information. We found that the effect of gesture in directing attention away from the video was smaller for the TBI group than the NC group (Node 1), and the effect of gesture in facilitating fixations away from distractors and towards verb-relevant pictures was nonsignificant for the TBI group (Node 2). However, gesture was similarly effective for TBI and NC participants at driving fixations away from the competitor and towards the target (Node 3). Thus, the reduced sensitivity to gesture in the TBI group appears to be due to participants with TBI looking more at the video and distractor pictures relative to verb-relevant pictures. When participants with TBI were looking at verbrelevant pictures, the relative probability of looking at the target compared to the competitor did not differ in magnitude from the NC group. This suggests that the reduced benefit of gesture in the TBI group is not due to a deficit in comprehending gesture, but rather in the misallocation of attention to relevant and irrelevant stimuli in multimodal contexts during on-line language processing.

Such difficulties in processing multimodal language may underlie the communication difficulties that people with TBI experience in rich communication contexts in real-world social interaction. Trujillo and Holler (2023) proposed that multimodal language processing includes two processes: 1) the segregation of the relevant signals from irrelevant information and 2) the contextual binding of co-occurring signals. The observed attenuation in speech-gesture integration in TBI could result from differences at either level; however, the results of the dynamic tree analysis are more consistent with a deficit in segregation. Although participants with TBI showed intact benefits of gesture in fixating the target vs. the competitor at Node 3, their reduced effect of gesture at Nodes 1 and 2 suggests that they may have difficulty with rapidly dismissing irrelevant cues (e.g., grooming movements, distractor items). Relevant communication signals can occur in both verbal (e.g., speech, vocal prosody, pause duration or dysfluency) and visual channels (e.g., gesture, eye gaze, blinks, nods, eyebrow and mouth movements, facial expression, body posture). However, these channels can also contain non-communicative information (e.g., coughing or clearing one’s throat, grooming movements, adjusting body position for comfort, glancing or nodding at a passerby). Therefore, both noncommunicative and communicative signals are embedded in an interaction, requiring the listener to quickly filter irrelevant information while binding meaningful information across modalities (see Trujillo & Holler, 2023 for a theoretical framework). A disruption in segregating relevant and irrelevant information could explain some of the social communication difficulties adults with TBI experience in rich contexts. This is the first study of multimodal language processing in-the-moment in TBI, and more studies are needed to confirm this hypothesis and to improve our understanding of the mechanisms underlying social communication difficulties in TBI.

Examining significant effects of trial number provides insight into learning processes over the course of the experiment. At both Nodes 1 and 3, we found that the benefit of gesture for increasing fixations to relevant stimuli increased over the course of the experiment. In other words, participants learned that the gesture was a meaningful clue (or that grooming movements were not) and showed stronger effects of movement type as the experiment progressed. The lack of three-way interactions with movement type, trial number, and group indicated that this was true for both NC and TBI groups. In addition, we found that the probability of looking away from the video toward pictures decreased over the course of the experiment for NC participants. This likely reflects learning over time and habituation to aspects of the pictured items (e.g., pictures appear in repeated closed sets, pictures occur in semantically related pairs) and the video stimulus (e.g., sometimes the speaker’s movement is helpful and sometimes it is not). This learning effect was stronger in the TBI group. Visual analysis revealed that this was driven by a higher proportion of picture fixations relative to video fixations in the TBI group than NC group in early trials, resulting in a greater change in the relative probability of picture-to-video fixations across the 240 trials in the TBI group. We speculate that participants with TBI required more trials to learn the relative importance of various visual cues in the experiment. This has important implications for understanding how people with TBI weight different sources of information in multimodal communication contexts that provide co-occurring or competing visual cues and may further support a difficulty in filtering out irrelevant cues in the TBI group.

More generally, our findings demonstrate the utility of eye-tracking as a sensitive tool to detect disruptions in language processing in TBI. Although participants with TBI demonstrated high accuracy in their click responses to the target items, their fixations reveal evidence of reduced on-line speech-gesture integration. Studies of other clinical populations, including children with specific language impairment and hearing loss, have shown a similar pattern of results in which eye-tracking uniquely reveals language processing differences, despite accurate behavioral responses (Klein, Walker, & McMurray, 2023; McMurray, Samelson, Lee, & Bruce Tomblin, 2010). This has important clinical implications for identifying language deficits after TBI. Assessments that isolate spoken language and focus only on behavioral accuracy may miss disruptions to language processing in-the-moment and fail to characterize patients’ abilities to use and process language in context. Indeed, current assessment practices lack sensitivity to detect communication deficits in TBI (Barwood & Murdoch, 2013; Blyth, Scott, Bond, & Paul, 2012), necessitating a shift to more ecologically valid and multimodal language assessments. Critically, the attenuation in gesture benefit identified in the current study is likely to scale up in more complex communication contexts. In this case, gestures were embedded in a predictable carrier phrase, and the gestures were large, iconic, and highly salient. In everyday language use, sentences are not produced one at a time, but rather are delivered in a continuous incremental stream of linguistic input that builds on discourse history and accumulates across turns of an interaction. It is possible that the benefit of gesture might suffer additional reductions in more dynamic or interactive contexts such as dyadic or group conversation or with the layering of additional social and cognitive demands. We discuss further implications for this research below.

Despite the attenuated effect of gesture in the TBI group, gesture did facilitate their fixations to the target item, showing evidence of on-line speech-gesture integration in TBI. This complements a previous finding by our group (Clough, Padilla, et al., 2023) that adults with TBI also successfully integrate unique information from gesture into their comprehension of and memory for stories. This converging evidence from behavioral and eye-tracking methods builds a foundation for understanding the communicative abilities of adults with TBI from a multimodal language perspective, taking into consideration the fact that real-world communication occurs in rich environments that contain a variety of social cues, including gesture and visual context. However, the current study represents a controlled experimental paradigm in which the role of gesture in language processing was isolated from other communicative cues. In addition to demonstrating evidence of successful gesture comprehension, adults with TBI also show intact perception of other social cues when studied in isolation, including eye-gaze (Mutlu, Duff, & Turkstra, 2019), interpersonal distance (Mutlu et al., 2019), and disfluencies (Diachek, Brown-Schmidt, & Duff, 2024). The finding that adults with TBI show successful social cue perception in isolation suggests that the fundamental building blocks of language processing and social communication are available to them. However, little is known about how both adults with and without TBI weight and integrate co-occurring information from multiple social cues during language processing. Studying the combinatorial effect of these cues in TBI may reveal unique insights into when and how communication breakdowns occur in the kinds of rich multimodal communication contexts that characterize everyday life. It is possible that social communication impairments in TBI might arise from disruptions to the rapid integration of multiple cues across speakers, modalities, and time. In particular, the hallmark diffuse axonal injury and overall reduced connectivity of neural pathways may disrupt multimodal processing and integration (Hayes et al., 2016; McDonald et al., 2019).

This is the first study of on-line speech-gesture integration in individuals with TBI and one of only a few studies to examine gesture comprehension in TBI (Bara et al., 2001; Clough, Padilla, et al., 2023; Evans & Hux, 2011), addressing a clear gap in our understanding of language processing after TBI (Clough & Duff, 2020). The benefits of gesture for comprehension and memory are well documented in neurotypical individuals (Dargue, Sweller, & Jones, 2019; Hostetter, 2011), yet it is unclear whether these benefits extend to adults with TBI. Further, very little is known about how adults with TBI use gesture in spontaneous language production. There is a large literature supporting evidence for selforiented cognitive functions of gesture for the speaker (Kita, Alibali, & Chu, 2017), providing additional motivation to increase the study of gesture in adults with TBI who can present with deficits across cognitive domains (e.g., memory, attention, executive function, working memory, perception, language). It remains an open empirical and clinically imperative question whether gesture can be leveraged to improve communication and cognition in individuals with TBI.

In the current study, we demonstrate feasibility and utility in using gaze as a window into the cognitive-linguistic processes of adults with TBI as they unfold in real time. Participants with TBI were calibrated successfully and were able to sustain position in the chin rest for the duration of the 45-min eye-tracking experiment. The current study also revealed many insights into methodological considerations for future studies examining multimodal communication with eyetracking. Although many participants in both groups shifted their gaze to objects during the critical analysis window, others remained fixated on the video until it disappeared from view (see supplementary materials for individual participant effects), potentially reducing our ability to detect measures of semantic integration of gesture in these individuals. The video stimuli were a crucial component of the current study’s multimodal design, expanding on other implementations of the visual world scene which have largely examined gaze in response to auditory stimuli only. The use of video stimuli more accurately reflects the dynamic language processing demands that characterize face-to-face communication in which the listener must integrate multiple meaningful channels of information while also filtering out irrelevant stimuli. However, to reduce the impact of the strong attentional capture of the video, future studies might consider having the video disappear after critical information in gesture has been produced or creating stimuli that provide meaningful information in the gesture modality only without subsequent disambiguation in speech (e.g., “The girl will eat the very good food” with a *sandwich-holding* gesture). There are many other potential avenues for studying speech-gesture integration in rich visual contexts. For example, the current eye-tracking study included picture options in the four corners, but others might include more elaborate visual scenes in which the speaker is integrated into the visual context. Yet another possibility would be to use virtual reality and avatars to simulate real-world environments in a virtual world paradigm which can be combined with eye-tracking (Peeters, 2019). Future elaborations on these designs have the potential to provide unique insights into how people direct attention and integrate information across social cues in rich multimodal communication contexts.

Finally, although we show evidence that adults with TBI as a group have reduced speech-gesture integration in multimodal language processing, there are likely individual differences in cognitive or neuroanatomical profile that moderate speech-gesture integration abilities. For example, in neurotypical people, working memory is posited to be a key cognitive resource in speech-gesture integration (Özer & Göksun, 2020a) with many studies reporting modest relationships between working memory and gesture comprehension (Aldugom et al., 2020; Wu & Coulson, 2014, 2015; Özer & Göksun, 2020b). In contrast with these studies, our exploratory analysis using the List Sorting Working Memory subtest of the NIH Toolbox Cognitive Battery showed weak evidence for a relationship between working memory and effects of gesture on target fixations in both TBI and NC subgroups. This analysis was limited by having only one measure of (verbal) working memory and a reduced sample size given that some participants did not complete neuropsychological testing. This line of inquiry would benefit from comprehensive testing across cognitive domains as well as multiple measures of speech-gesture integration to better understand how disruptions to working memory and other cognitive domains (or overall deficit severity more generally) impede speech-gesture integration and contextualized language processing in individuals with TBI. Neural correlates of speech-gesture integration have been identified across the left frontal-posterior temporal network (for a review, see Kandana Arachchige, Simoes Loureiro, Blekic, Rossignol, & Lefebvre, 2021; [Özyürek, 2014). Further, a growing literature links variation in participants’ anticipatory eye movements to both verbal and nonverbal factors in healthy adults; indeed, vocabulary size, verbal fluency, language experience and responsiveness to spatial cuing have been shown to predict anticipatory eye fixation behavior (Hintz, Meyer, & Huettig, 2017; James, Minnihan, & Watson, 2023; Rommers, Meyer, & Huettig, 2015), and unique language and literacy skills may differentially predict the ability to activate predictable outcomes and inhibit implausible or irrelevant outcomes (Kukona et al., 2016). Given the diffuse nature of grey and white matter injury in TBI and the inherent heterogeneity in cognitive profiles (Covington & Duff, 2021), studying individual differences in speech-gesture integration in TBI has the potential to yield additional insights into the mechanisms supporting multimodal communication. We do not expect that speech-gesture integration would be uniformly disrupted across individuals with TBI. Examining the factors that predict successful speech-gesture integration is an important future direction for identifying people who are most at risk for multimodal language processing deficits and subsequently informing assessment and personalized treatment practices to improve the communicative lives of adults with TBI. This study advances our understanding of the communicative abilities of adults with TBI and could lead to a more mechanistic account of the communication difficulties adults with TBI experience in rich real-world communication contexts that require the processing and integration of multiple cooccurring cues. The findings highlight the importance of increasing the ecological validity of language assessment and continued efforts to identify the cognitive and neural mechanisms that support multimodal language processing.

## Supplementary Material

supp

Supplementary data to this article can be found online at https://doi.org/10.1016/j.cortex.2024.08.008.

## Figures and Tables

**Table 1 –:** Descriptive statistics for TBI and NC groups.

	Sex (*n*)		Age (years)			Edu (years)			TSO (months)		
	Male	Female	Mean (SD)	Min	Max	Mean (SD)	Min	Max	Mean (SD)	Min	Max
TBI	18	27	38.76 (9.99)	24	55	14.60 (2.65)	11	20	58.42 (56.22)	6	248
NC	19	25	37.93 (10.67)	20	55	14.73 (2.59)	12	20	NA	NA	NA

*Note.* TBI = traumatic brain injury. NC = non-injured comparison. Education (Edu) reflects years of highest degree obtained. Time since onset (TSO) is presented in months.

**Table 2 –:** Demographic and injury information for participants with TBI.

ID	Age	Edu	Etiology	TSO	LOC	Neuroimaging	GCS	PTA
5003	31–35	18	Ped vs. auto	69	N/A	SDH	11	>24 h
5014	51–55	16	MVA	228	LOC >30 min	N/A	N/A	>24 h
5016	21–25	16	MVA	61	LOC >30 min	SAH	13	>24 h
5018	41–45	18	MVA	194	LOC >30 min	SAH	3	>24 h
5019	46–50	16	Ped vs. auto	75	N/A	SAH; SDH	6	>24 h
5021	41–45	18	MVA	74	LOC >30 min	EDH; SAH	3	>24 h
5029	36–40	14	Non-motorized vehicle accident	55	LOC <30 min	SDH; IPH; SAH	14	<24 h
5034	36–40	16	MVA	77	LOC >30 min	SAH	3	>24 h
5040	41–45	12	MVA	117	LOC >30 min	SDH; SAH; uncal herniation	3	>24 h
5041	31–35	16	MVA	98	No LOC	No acute intracranial findings	10	>24 h
5046	46–50	18	Non-motorized vehicle accident	88	LOC <30 min	SAH	14	>24 h
5050	31–35	18	Ground-level fall	57	LOC >30 min	SAH; IPH	15	<24 h
5051	51–55	16	MVA	42	LOC <30 min	SAH; SDH	14	<24 h
5052	31–35	14	MVA	44	LOC <30 min	SDH; SAH	9	>24 h
5058	36–40	12	MCC	152	LOC <30 min	SAH; SDH; PCH	8	>24 h
5086	36–40	16	Ped vs. auto	131	LOC >30 min	SAH	15	<24 h
5095	41–45	12	Other	69	LOC >30 min	ICH; parenchymal contusions, SAH; SDH	3	>24 h
5104	36–40	20	Struck by object	49	LOC <30 min	SDH; scattered SAH; right temporal hemorrhage	15	<24 h
5108	41–45	12	MVA	44	LOC >30 min	Bilateral SAH	3	>24 h
5111	26–30	16	MVA	84	LOC <30 min	Shear Injury; DAI		>24 h
5118	26–30	18	MVA	63	LOC >30 min	SDH	10	>24 h
5119	36–40	16	MVA	248	LOC >30 min	SAH; Possible right frontal contusion	N/A	>24 h
5122	51–55	18	Non-motorized vehicle accident	41	LOC <30 min	SAH	15	>24 h
5123	51–55	12	MCC	41	LOC <30 min	IPH; SDH; SAH	14	>24 h
5125	51–55	12	Ground-level fall	29	No LOC	SDH; SAH	15	No
5126	46–50	12	MVA	44	LOC >30 min	SDH	3	>24 h
5129	51–55	12	Other	27	LOC <30 min	SDH; SAH	12	<24 h
5131	41–45	12	MVA	27	LOC >30 min	SDH	12	>24 h
5137	26–30	16	Ped vs. auto	24	LOC >30 min	EDH; SDH: SAH	3	>24 h
5141	26–30	12	MVA	21	LOC >30 min	SDH	13	<24 h
5156	51–55	12	MVA	54	LOC >30 min	SDH	15	No
5158	31–35	16	MVA	18	LOC <30 min	SAH	15	N/A
5161	26–30	12	MVA	14	LOC >30 min	SDH; PCH; DAI	10	>24 h
5164	46–50	16	Fall from height	16	LOC >30 min	SDH	3	>24 h
5165	21–25	12	Other	19	LOC >30 min	SDH; SAH	8	>24 h
5166	31–35	12	MVA	14	LOC >30 min	SAH	7	>24 h
5168	21–35	12	MVA	13	N/A	SAH	14	>24 h
5169	51–55	20	Non-motorized vehicle accident	18	LOC >30 min	SDH; SAH	N/A	<24 h
5174	41–45	16	Ped vs. auto	19	LOC >30 min	DAI; SAH; IVH; cerebral hematoma	3	>24 h
5175	31–35	16	Ground-level fall	11	N/A	SDH; SAH; bifrontal contusions	15	>24 h
5176	26–30	12	MCC	6	LOC >30 min	Shear/DAI	7	>24 h
5178	26–30	12	MVA	15	LOC >30 min	IPH; SAH; IVH; DAI	3	>24 h
5179	26–30	12	Ped vs. auto	13	No LOC	IPH; SDH; SAH; hemorrhagic contusions	15	<24 h
5182	36–40	12	Ground-level fall	16	LOC <30 min	SAH; SDH, hemorrhagic contusions, PCH	13	<24 h
5183	31–35	11	MCC	10	LOC <30 min	Hemorrhagic contusions; extra-axial hemorrhage; SAH	14	>24 h

*Note.* ID = participant ID number. Education (Edu) reflects years of highest degree obtained. MVA = motor vehicle accident. MCC includes both motorcycle and snowmobile accidents. Non-motor = non-motorized vehicle accident. Ped vs. auto = participant was hit by car while walking or running. Time since onset (TSO) is presented in months. Loss of consciousness (LOC) is presented in minutes. SDH = subdural hematoma. SAH = subarachnoid hemorrhage. IPH = intraparenchymal hemorrhage. IVH = intraventricular hemorrhage. ICH = intracranial hemorrhage. EDH = epidural hematoma. DAI = diffuse axonal injury. PCH = parenchymal hemorrhage. Glasgow Coma Scale (GCS) is total score at time of first post-injury measurement. PTA = post-traumatic amnesia. N/A = information was not available.

**Table 3 –:** Mean NIH toolbox cognition battery scores.

Group	Dimensional Change Card Sort *(SD)*	Flanker Inhibitory Control & Attention *(SD)*	Picture Sequence Memory *(SD)*	List Sorting Working Memory *(SD)*	Pattern Comparison *(SD)*	Fluid Composite *(SD)*	Picture Vocabulary *(SD)*	Oral Reading Recognition *(SD)*	Crystallized Composite *(SD)*	Total Cognition *(SD)*
TBI (n = 42)	95.57 (19.44)	79.48 (14.34)	106.67 (15.92)	104.62 (15.90)	92.67 (20.32)	93.43 (19.12)	102.29 (12.54)	102.75 (12.16)	102.30 (12.22)	97.15 (15.15)
NC (n = 26)	109.15 (14.58)	93.15 (14.19)	122.88 (17.90)	113.88 (14.29)	114.81 (19.46)	116.08 (14.65)	116.73 (11.92)	116.58 (13.56)	118.15 (12.79)	119.85 (10.44)
t-test	*p* = .003	*p* < .001	*p* < .001	*p* = .02	*p* < .001	*p* < .001	*p* < .001	*p* < .001	*p* < .001	*p* < .001

*Note.* Scores reported are age-corrected Standard Scores (*M* = 100, *SD* = 15). Fluid Composite includes Dimensional Change Card Sort, Flanker Inhibitory Control and Attention, Picture Sequence Memory, List Sorting Working Memory, and Pattern Comparison tests. Crystallized Composite includes Picture Vocabulary and Oral Reading Recognition tests.

**Table 4 –:** Results of dynamic GLMM for participants with TBI (n = 45) and non-injured participants (n = 44), 240 trials and 5,361,360 observations.

Fixed Effects	Estimate	SE	z-value	*p*-value
(Intercept)	−1.877	.098	−19.212	<.001
Movement (grooming = −.5, gesture = .5)	.594	.066	8.938	<.001
Group (NC = 0, TBI = 1)	.150	.102	1.472	.141
AR1	11.325	.125	90.899	<.001
Time	.512	.016	32.423	<.001
Trial Number	−.038	.015	−2.536	.011
Movement*Group	−.173	.083	−2.086	.037
Movement*Trial Number	.114	.030	3.773	<.001
Group*Trial Number	−.025	.021	−1.182	.237
Movement*Group*Trial Number	−.045	.042	−1.065	.287
*Random Effects*	*Variance*		*SD*	
Participant (intercept)	.577		.759	
AR(1) slope by participant	1.327		1.152	
Movement slope by participant	.083		.289	
Item (intercept)	.009		.097	

*Note.* NC group is dummy coded as the reference level.

**Table 5 –:** Results of Dynamic Tree-based Item-response Model for Participants with TBI (n = 45) and Non-injured Participants (n = 44) in the Node 1 (5,361,360 observations), Node 2 (752,853 observations), and Node 3 analyses (439,361 observations).

Fixed Effects	Estimate	SE	z-value	*p*-value
node1 intercept	−1.200	.101	−11.822	<.001
node1*Movement	.234	.035	6.681	<.001
node1*Group	.154	.108	1.422	.155
node1*Trial Number	−.001	.000	−5.259	<.001
node1*Time	.004	.000	41.435	<.001
node1*AR1	10.658	.119	89.696	<.001
node1*Movement*Group	−.094	.047	−1.982	.047
node1*Movement*Trial Number	.001	.000	3.483	<.001
node1*Group*Trial Number	−.001	.000	−4.015	<.001
node1*Movement*Group*Trial Number	−.001	.000	−1.405	.160
node2 intercept	.413	.073	5.656	<.001
node2*Movement	.274	.052	5.225	<.001
node2*Group	−.156	.094	−1.654	.098
node2*Trial Number	.000	.000	−.092	.927
node2*Time	.002	.000	6.194	<.001
node2*AR1	10.108	.092	109.606	<.001
node2*Movement*Group	−.190	.072	−2.649	.008
node2*Movement*Trial Number	.000	.001	−.663	.508
node2*Group*Trial Number	.000	.000	−.023	.982
node2*Movement*Group*Trial Number	.001	.001	1.223	.221
node3 intercept	.305	.053	5.762	<.001
node3*Movement	.989	.119	8.317	<.001
node3*Group	−.138	.072	−1.904	.057
node3*Trial Number	.001	.001	1.284	.199
node3*Time	.001	.001	1.036	.300
node3*AR1	11.792	.162	73.011	<.001
node3*Movement*Group	−.218	.156	−1.397	.162
node3*Movement*Trial Number	.003	.001	2.570	.010
node3*Group*Trial Number	.001	.001	.926	.354
node3*Movement*Group*Trial Number	−.001	.001	−.761	.446
*Random Effects*	*Variance*		*SD*	
Participant (node1 intercept)	.621		.788	
Participant (node2 intercept)	.158		.397	
Participant (node3 intercept)	.051		.227	
AR1 slope by participant at node1	1.208		1.099	
AR1 slope by participant at node2	.607		.779	
AR1 slope by participant at node3	1.834		1.354	
Movement slope by participant at node1	.028		.167	
Movement slope by participant at node2	.036		.189	
Movement slope by participant at node3	.323		.568	
Item (node1 intercept)	.004		.067	
Item (node2 intercept)	.019		.137	
Item (node3 intercept)	.000		.020	

*Note.* NC group is dummy coded as the reference level.

## Footnotes

1 Aligning the start of the critical analysis window to the onset of the movement stroke captures participants’ fixations from the moment participants receive meaningful information about the target item in the gesture condition. Due to the requirement of dynamic GLMM models to have the same size analysis window for all trials, we set the end of the analysis window to the average onset of the target noun in speech (2700 msec later). This results in some variability in how much information participants received about the target word in speech at the end of the analysis window across trials. To ensure effects were not driven by early information about the target word in speech on some of the trials, we reanalyzed the data setting the end of the critical analysis window to the earliest onset of the target word in speech for 95% of trials (2155 msec later). In this truncated analysis, the movement type by group interaction was marginal ( *β* = −.18, *z* = −1.78, *p* = .07). It should be noted that in the original analysis reported above, the timing of target word onsets across movement type conditions and participant groups are equivalent. Thus, any observed group and condition effects are driven by those conditions rather than the timing of the target word in speech. Detailed results of this truncated analysis can be found in supplementary materials (Supplementary Analysis 1).
