# Supplementary material for: Reduced on-line speech gesture integration during multimodal language processing in adults with moderate-severe traumatic brain injury: Evidence from eye-tracking
Source: Cortex. Author manuscript; Available in PMC 2026 May 17. (PMC13180290; doi:10.1016/j.cortex.2024.08.008)
Supplement: supp [file NIHMS2166636-supplement-supp.docx]

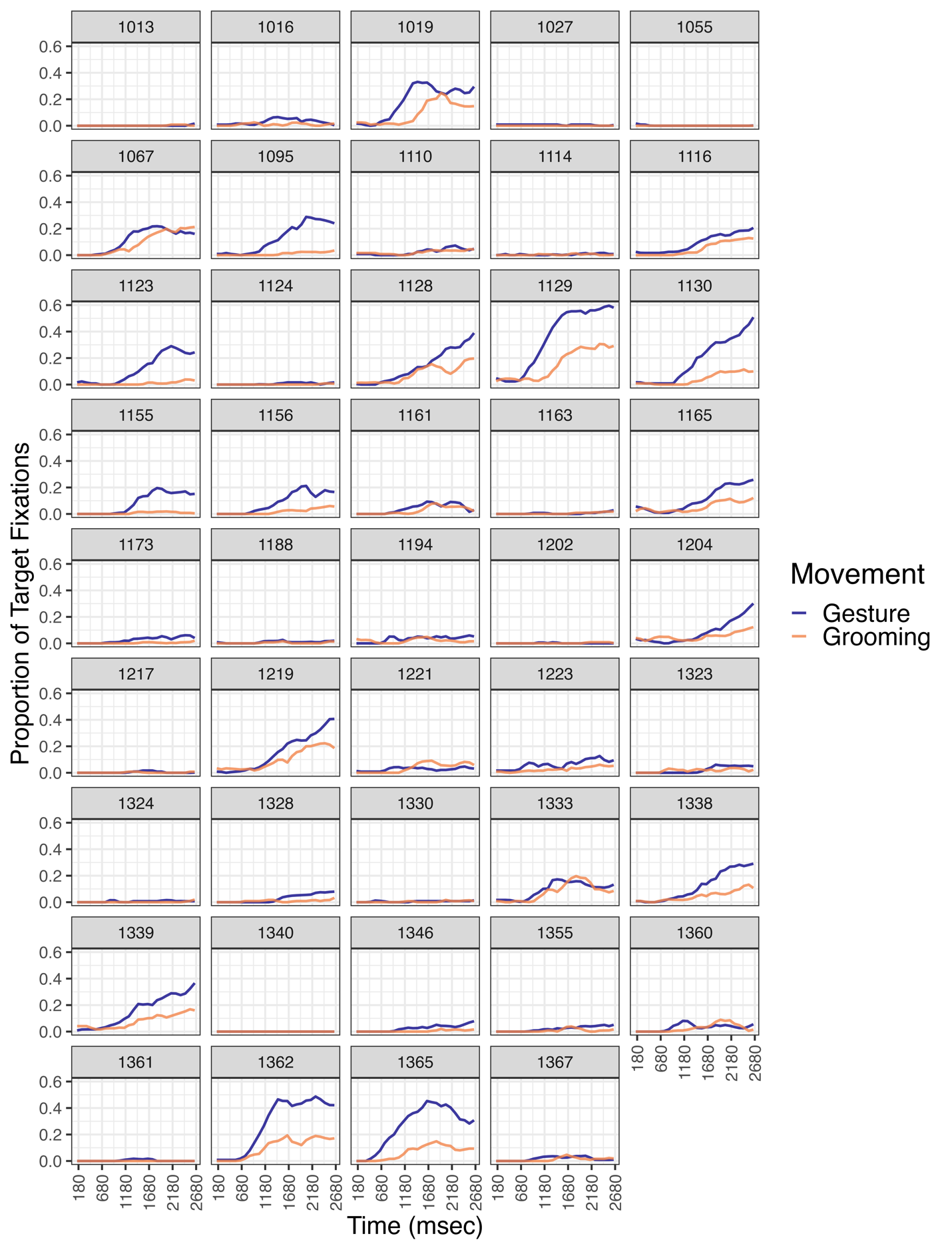


**Figure SI 1**. *Individual gesture effects for the 44 participants in the NC group.*


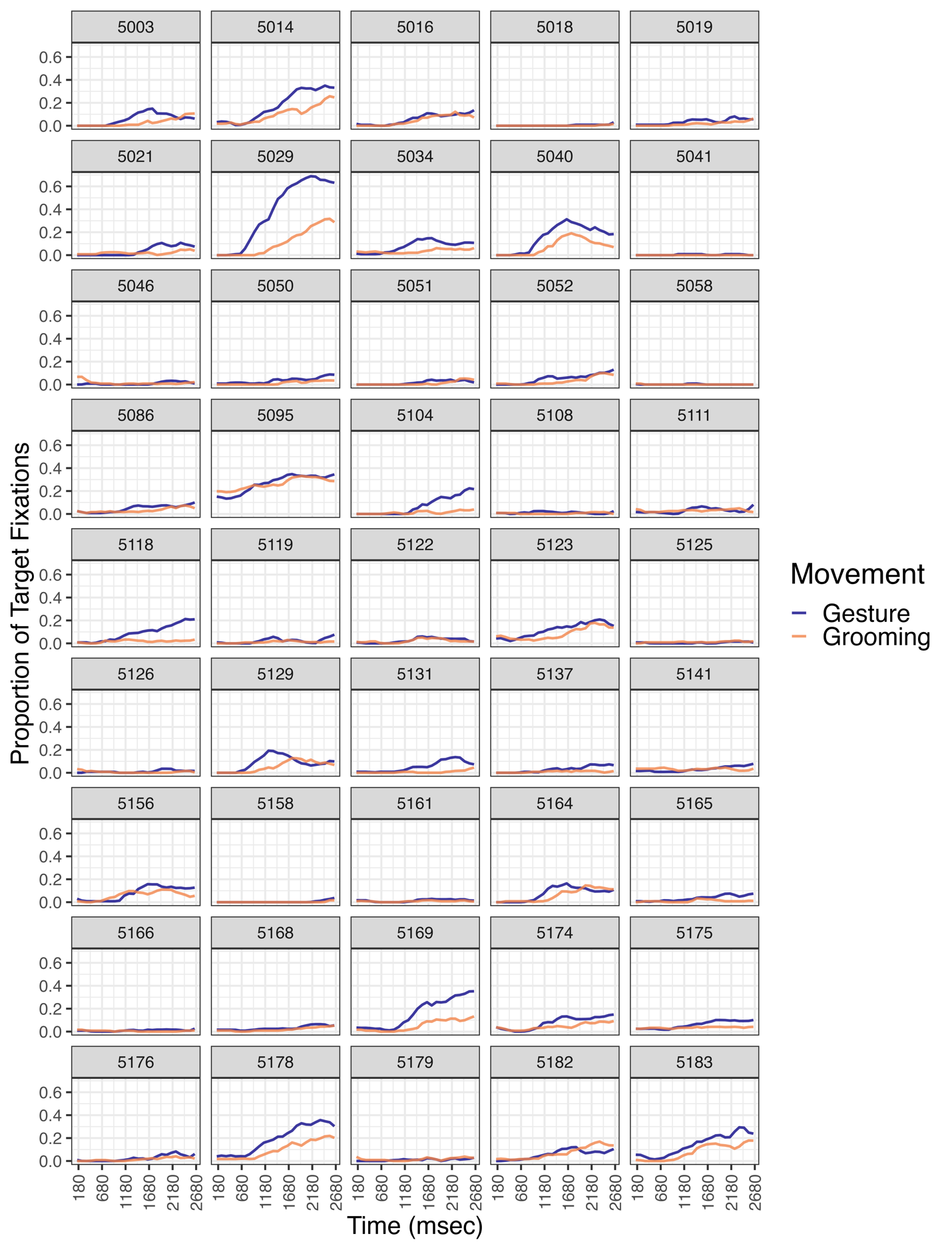


**Figure SI 2.** *Individual gesture effects for the 45 participants in the TBI group.*


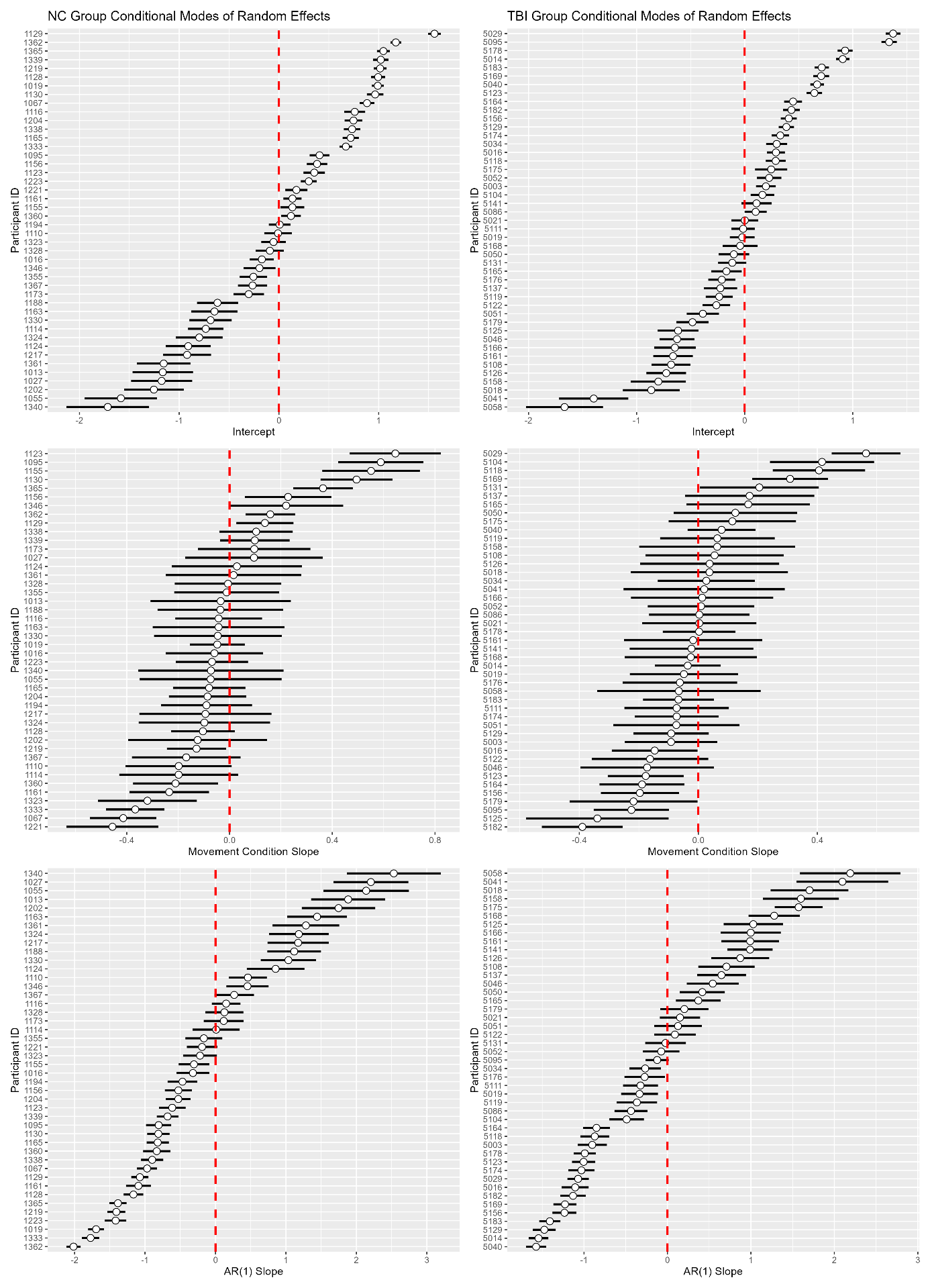


**Figure SI 3.** *Individual differences in predicted random effects.*

**Supplementary Analysis 1: Analysis of binary fixations to target item with shortened critical analysis window**

Our primary analysis modeled the probability of binary fixations to the target item as a function of participant group, movement type, trial number, and their interaction with covariates for Time and AR1 across the critical analysis window which began at onset of movement stroke and ended at the average onset of the target item in speech. Due to reviewer concerns that variability in stimulus item timing results in participants receiving some information about the target word in speech during this analysis window, we re-analyzed the data with a shortened critical analysis window, setting the end of the window to the earliest onset of the target word in speech for 95% of the trials, which occurred 2155 msec after the movement stroke. Thus, this analysis captures interpretation of the head noun, verb, and movement, prior to most noun information. Note that the timing of movement strokes and target nouns are equivalent across movement conditions and participant groups. Results of the dynamic GLMM model parallel the primary model reported in the main text and are presented in SI Table 2.

There was a significant effect of movement type ($\hat{\beta}$ = 0.64, *z* = 7.57, *p* < .001); NC participants were 1.89 times more likely to fixate the target item during the shortened analysis window when the speaker produced a meaningful gesture compared to a meaningless grooming movement. There was a significant effect of group ($\hat{\beta}$ = 0.22, *z* = 2.00, *p* = 0.05), indicating that participants with TBI were more likely to fixate the target item across all trials during the shortened analysis window. The group*movement type interaction was not significant ($\hat{\beta}$ = -0.18, *z* = -1.78, *p* = .07), suggesting that the effect of movement type was smaller in the TBI group relative to the NC group.

There was a significant effect of trial number on probability of fixations to the target ($\hat{\beta}$ = -0.05, *z* = -2.65, *p* = .008); across both grooming and gesture trials, the probability of fixating the target item during the critical window decreased over the course of the experiment for non-injured participants. A lack of significant interaction between group and trial number ($\hat{\beta}$ = -0.04, *z* = -1.41, *p* = .16) indicated that the magnitude of effect was not significantly different for participants with TBI. There was a significant interaction between movement type and trial number ($\hat{\beta}$ = 0.11, *z* = 2.97, *p* = .003), where the positive effect of gesture on the probability of target fixations increased across trials of the experiment. There was no three-way interaction between movement type, trial number, and group ($\hat{\beta}$ = -0.02, *z* = -0.39, *p* = .70). The significant effect of time ($\hat{\beta}$ = 0.83, *z* = 34.45, *p* < .001) reflects the increasing probability of target fixations over time within a trial, and the significant effect of AR1 ($\hat{\beta}$ = 11.42, *z* = 81.40, *p* < .001) reflects the serial dependency from time-point to time-point in whether or not participants fixated the target at a given time point.

| **SI Table 1.** *Results of Dynamic GLMM for Participants with TBI (n=45) and Non-injured Participants (n=44), 240 Trials and 5,361,360 Observations.* | | | | |
| --- | --- | --- | --- | --- |
| *Fixed Effects* | *Estimate* | *SE* | *z-value* | *p-value* |
| (Intercept) | -2.068 | 0.108 | -19.206 | <0.001 |
| Movement  (grooming = -0.5, gesture = 0.5) | 0.637 | 0.084 | 7.572 | <0.001 |
| Group (NC = 0, TBI = 1) | 0.224 | 0.112 | 2.004 | 0.045 |
| AR1 | 11.418 | 0.140 | 81.403 | <0.001 |
| Time | 0.827 | 0.024 | 34.450 | <0.001 |
| Trial Number | -0.048 | 0.018 | -2.648 | 0.008 |
| Movement*Group | -0.184 | 0.103 | -1.782 | 0.075 |
| Movement*Trial Number | 0.107 | 0.036 | 2.969 | 0.003 |
| Group*Trial Number | -0.036 | 0.026 | -1.409 | 0.159 |
| Movement*Group*Trial Number | -0.020 | 0.051 | -0.391 | 0.696 |
| *Random Effects* | *Variance* | | *SD* | |
| Participant (intercept) | 0.634 | | 0.796 | |
| AR(1) slope by participant | 1.537 | | 0.124 | |
| Movement slope by participant | 1.220 | | 0.349 | |
| Item (intercept) | 0.010 | | 0.102 | |
| *Note.* NC group is dummy coded as the reference level. | | | | |

| **SI Table 2.** *Results of Dynamic GLMM for Participants with TBI (n=45) and Non-injured Participants (n=44), 240 Trials and 5,361,360 Observations.* | | | | |
| --- | --- | --- | --- | --- |
| *Fixed Effects* | *Estimate* | *SE* | *z-value* | *p-value* |
| (Intercept) | -1.727 | 0.097 | -17.859 | <0.001 |
| Movement  (grooming = -0.5, gesture = 0.5) | 0.421 | 0.060 | 7.029 | <0.001 |
| Group (TBI = 0, NC = 1) | -0.150 | 0.103 | -1.455 | 0.146 |
| AR1 | 11.325 | 0.127 | 89.367 | <0.001 |
| Time | 0.512 | 0.016 | 32.413 | <0.001 |
| Trial Number | -0.064 | 0.015 | -4.264 | <0.001 |
| Movement*Group | 0.173 | 0.083 | 2.082 | 0.037 |
| Movement*Trial Number | 0.069 | 0.030 | 2.316 | 0.021 |
| Group*Trial Number | 0.025 | 0.021 | 1.182 | 0.237 |
| Movement*Group*Trial Number | 0.045 | 0.042 | 1.065 | 0.287 |
| *Random Effects* | *Variance* | | *SD* | |
| Participant (intercept) | 0.577 | | 0.759 | |
| AR(1) slope by participant | 1.327 | | 1.152 | |
| Condition slope by participant | 0.083 | | 0.289 | |
| Item (intercept) | 0.009 | | 0.097 | |
| *Note*. TBI group is dummy coded as the reference level. | | | | |

| **SI Table 3.** *Results of Dynamic Tree-based Item-response Model for Participants with TBI (n=45) and Non-injured Participants (n=44) in the Node 1 (5,361,360 observations), Node 2 (752,853 observations), and Node 3 analyses (439,361 observations).* | | | | |
| --- | --- | --- | --- | --- |
| *Fixed Effects* | *Estimate* | *SE* | *z-value* | *p-value* |
| node1 intercept | -1.048 | 0.100 | -10.451 | <0.001 |
| node1*Movement | 0.139 | 0.033 | 4.175 | <0.001 |
| node1*Group | -0.151 | 0.108 | -1.395 | 0.163 |
| node1*Trial Number | -0.002 | 0.000 | -11.509 | <0.001 |
| node1*Time | 0.004 | 0.000 | 41.427 | <0.001 |
| node1*AR1 | 10.679 | 0.119 | 89.735 | <0.001 |
| node1*Movement*Group | 0.093 | 0.047 | 1.963 | 0.050 |
| node1*Movement*Trial Number | 0.000 | 0.000 | 1.660 | 0.097 |
| node1*Group*Trial Number | 0.001 | 0.000 | 4.019 | <0.001 |
| node1*Movement*Group*Trial Number | 0.001 | 0.000 | 1.409 | 0.159 |
| node2 intercept | 0.256 | 0.069 | 3.703 | <0.001 |
| node2*Movement | 0.085 | 0.049 | 1.738 | 0.082 |
| node2*Group | 0.158 | 0.094 | 1.676 | 0.094 |
| node2*Trial Number | 0.000 | 0.000 | -0.132 | 0.895 |
| node2*Time | 0.002 | 0.000 | 6.175 | <0.001 |
| node2*AR1 | 10.153 | 0.093 | 109.649 | <0.001 |
| node2*Movement*Group | 0.191 | 0.072 | 2.660 | 0.008 |
| node2*Movement*Trial Number | 0.001 | 0.001 | 1.077 | 0.281 |
| node2*Group*Trial Number | 0.000 | 0.000 | 0.010 | 0.992 |
| node2*Movement*Group*Trial Number | -0.001 | 0.001 | -1.225 | 0.220 |
| node3 intercept | 0.176 | 0.051 | 3.435 | 0.001 |
| node3*Movement | 0.781 | 0.114 | 6.849 | <0.001 |
| node3*Group | 0.135 | 0.073 | 1.856 | 0.063 |
| node3*Trial Number | 0.001 | 0.000 | 2.658 | 0.008 |
| node3*Time | 0.001 | 0.001 | 1.049 | 0.294 |
| node3*AR1 | 11.854 | 0.161 | 73.574 | <0.001 |
| node3*Movement*Group | 0.215 | 0.156 | 1.374 | 0.169 |
| node3*Movement*Trial Number | 0.002 | 0.001 | 1.552 | 0.121 |
| node3*Group*Trial Number | -0.001 | 0.001 | -0.910 | 0.363 |
| node3*Movement*Group*Trial Number | 0.001 | 0.001 | 0.754 | 0.451 |
| *Random Effects* | *Variance* | | *SD* | |
| Participant (node1 intercept) | 0.620 | | 0.787 | |
| Participant (node2 intercept) | 0.159 | | 0.398 | |
| Participant (node3 intercept) | 0.052 | | 0.227 | |
| AR1 slope by participant at node1 | 1.211 | | 1.101 | |
| AR1 slope by participant at node2 | 0.612 | | 0.782 | |
| AR1 slope by participant at node3 | 1.817 | | 1.348 | |
| Movement slope by participant at node1 | 0.028 | | 0.167 | |
| Movement slope by participant at node2 | 0.035 | | 0.188 | |
| Movement slope by participant at node3 | 0.320 | | 0.566 | |
| Item (node1 intercept) | 0.004 | | 0.067 | |
| Item (node2 intercept) | 0.019 | | 0.137 | |
| Item (node3 intercept) | 0.000 | | 0.020 | |
| *Note.* TBI group is dummy coded as the reference level. | | | | |

**Supplementary Analysis 2: Cognitive predictors of speech-gesture integration**

A growing number of studies have linked co-speech gesture processing to working memory in neurologically healthy adults (Aldugom et al., 2020; Özer & Göksun, 2020b; Wu & Coulson, 2014, 2015). Using data available from the NIH Toolbox Cognitive Battery, we conducted an exploratory analysis to examine the role of working memory (list sorting subtest) in predicting target fixations across movement conditions in the TBI group. We modeled binary fixations to the target item as a function of movement type, trial number, working memory scores, and their interactions with covariates for Time and AR1 with random slopes for AR(1) and movement condition by participant and a random intercept for items. Results of the dGLMM model are presented in SI Table 4.

The effect of movement type was significant ($\hat{\beta}$ = 0.44, *z* = 7.65, *p* < 0.001). Participants with TBI were 1.55 times more likely to fixate the target item during the critical analysis window when the speaker produced a meaningful gesture compared to a meaningless grooming movement. There was no main effect of working memory on target fixations ($\hat{\beta}$ = -0.04, *z* = -0.24, *p* = 0.81). There was also no significant interaction between working memory and movement type ($\hat{\beta}$ = 0.14, *z* = 1.48, *p* = .14), suggesting that was no differentiation between participants across the range of working memory scores in the magnitude of the effect of gesture on target fixations.

There was a main effect of trial number on the probability of target fixations ($\hat{\beta}$ = -0.07, *z* = -4.21, *p* < .001); across both grooming and gesture movement trials, the probability of fixating the target item during the critical window decreased over the course of the experiment for TBI participants. There was no significant two-way interaction between trial number and working memory ($\hat{\beta}$ = 0.02, *z* = 0.72, *p* = .47). There was a significant interaction between movement type and trial number ($\hat{\beta}$ = 0.06, *z* = 2.01, *p* = .05), where the positive effect of gesture on the probability of target fixations increased across trials of the experiment. There was a significant three-way interaction between movement type, trial number, and working memory ($\hat{\beta}$ = 0.12, *z* = 2.07, *p* = .04). Visualizing the interaction (Figure SI 5) revealed that participants with TBI who had higher working memory scores showed a stronger condition effect (greater difference between gesture and grooming trials) at later trials of the experiment whereas those with lower working memory scores tended to show decreased condition effects across trials of the experiment.

The significant effect of time ($\hat{\beta}$ = 0.45, *z* = 18.95, *p* < .001) reflects the increasing probability of target fixations over time within a trial, and the significant effect of AR1 ($\hat{\beta}$ = 11.44, *z* = 64.86, *p* < .001) reflects the serial dependency from time-point to time-point in whether or not participants fixated the target at a given time point.

| **SI Table 4.** *Results of Dynamic GLMM examining effects of working memory for Participants with TBI (n=42), 240 Trials and 2,530,080 Observations.* | | | | |
| --- | --- | --- | --- | --- |
| *Fixed Effects* | *Estimate* | *SE* | *z-value* | *p-value* |
| (Intercept) | -1.742 | 0.104 | -16.830 | <0.001 |
| Movement  (grooming = -0.5, gesture = 0.5) | 0.436 | 0.057 | 7.647 | <0.001 |
| List Sorting Working Memory | -0.035 | 0.146 | -0.241 | 0.810 |
| AR1 | 11.436 | 0.176 | 64.864 | <0.001 |
| Time | 0.454 | 0.024 | 18.951 | <0.001 |
| Trial Number | -0.068 | 0.016 | -4.207 | <0.001 |
| Movement*List Sorting | 0.136 | 0.092 | 1.484 | 0.138 |
| Movement*Trial Number | 0.064 | 0.032 | 2.006 | 0.045 |
| List Sorting*Trial Number | 0.022 | 0.030 | 0.719 | 0.472 |
| Movement*List Sorting*Trial Number | 0.124 | 0.060 | 2.068 | 0.039 |
| *Random Effects* | *Variance* | | *SD* | |
| Participant (intercept) | 0.401 | | 0.634 | |
| AR(1) slope by participant | 1.175 | | 1.084 | |
| Condition slope by participant | 0.034 | | 0.184 | |
| Item (intercept) | 0.010 | | 0.100 | |


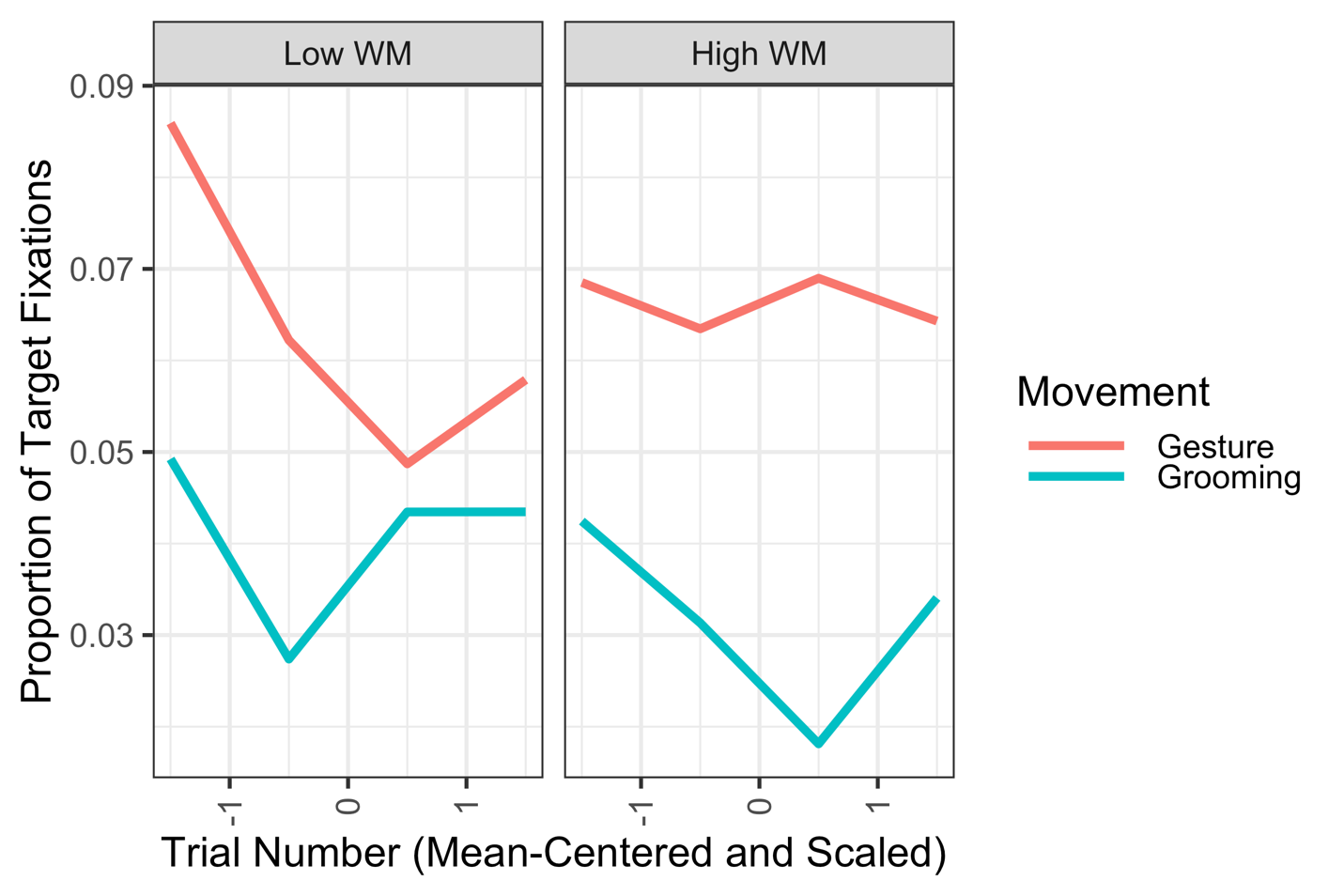


**Figure SI 5.** *Mean Proportion of Fixations to Target Item by Participants with TBI for Participants with Low and High Working Memory (WM) Scores (divided by the median) across 240 Trials of the Experiment (mean-centered and scaled).*

Because we only had working memory scores for 26/44 NC participants, we were underpowered to detect a three-way Group*Movement*Working Memory interaction. Therefore, by reviewer request, we ran a separate similar dGLMM model with just the n = 26 NC participants to determine whether there was a relationship between working memory scores and target fixations in the NC group. A boundary (singular) fit warning indicated that the variances for random effects were close to 0. Thus, we removed random slopes and included only random intercepts for participant and item. Full results of the model are shown in SI Table 5. There was no main effect of working memory in the NC group ($\hat{\beta}$ = 0.11, *z* = 0.24, *p* = 0.81). There was also no two-way interaction between working memory and movement condition ($\hat{\beta}$ = 0.10, *z* = 0.96, *p* = 0.34) and no three-way interaction between working memory, movement condition, and trial number ($\hat{\beta}$ = -0.17, *z* = -1.61, *p* = 0.11). There was a significant two-way interaction between working memory and trial number ($\hat{\beta}$ = 0.11, *z* = 2.18, *p* = 0.03), indicating that participants with higher working memory scores tended to increase target fixations across trials of the experiment relative to those with lower working memory scores.

| **SI Table 5.** *Results of Dynamic GLMM examining effects of working memory for Participants with NC (n=26), 240 Trials and 1,566,240 Observations.* | | | | |
| --- | --- | --- | --- | --- |
| *Fixed Effects* | *Estimate* | *SE* | *z-value* | *p-value* |
| (Intercept) | -2.000 | 0.221 | -9.010 | <0.001 |
| Movement  (grooming = -0.5, gesture = 0.5) | 0.647 | 0.048 | 13.563 | <0.001 |
| List Sorting Working Memory | 0.105 | 0.432 | 0.243 | 0.808 |
| AR1 | 10.565 | 0.041 | 257.508 | <0.001 |
| Time | 0.530 | 0.030 | 17.779 | <0.001 |
| Trial Number | -0.025 | 0.022 | -1.087 | 0.277 |
| Movement*List Sorting | 0.100 | 0.105 | 0.957 | 0.339 |
| Movement*Trial Number | 0.173 | 0.045 | 3.816 | <0.001 |
| List Sorting*Trial Number | 0.113 | 0.052 | 2.176 | 0.030 |
| Movement*List Sorting*Trial Number | -0.168 | 0.104 | -1.611 | 0.107 |
| *Random Effects* | *Variance* | | *SD* | |
| Participant (intercept) | 0.951 | | 0.975 | |
| Item (intercept) | 0.004 | | 0.067 | |
